# Multi-Omic Characterization of Epithelial–Mesenchymal Transition: Lipidomic and Metabolomic Profiles as Key Markers of TGF-β-Induced Transition in Huh7 Hepatocellular Carcinoma

**DOI:** 10.3390/cells14161233

**Published:** 2025-08-10

**Authors:** Agnese Bertoldi, Gaia Cusumano, Eleonora Calzoni, Husam B. R. Alabed, Roberto Maria Pellegrino, Sandra Buratta, Lorena Urbanelli, Carla Emiliani

**Affiliations:** 1 Department of Chemistry, Biology and Biotechnology, University of Perugia, Via del Giochetto, 06123 Perugia, Italy; agnese.bertoldi@dottorandi.unipg.it (A.B.); gaia.cusumano@dottorandi.unipg.it (G.C.); husambr.alabed@unipg.it (H.B.R.A.); roberto.pellegrino@unipg.it (R.M.P.); sandra.buratta@unipg.it (S.B.); lorena.urbanelli@unipg.it (L.U.); carla.emiliani@unipg.it (C.E.); 2 Centro di Digitalizzazione del Patrimonio Culturale (CeDiPa), University of Perugia, 06123 Perugia, Italy; 3 Centro di Eccellenza Materiali Innovativi Nanostrutturati (CEMIN), University of Perugia, Via del Giochetto, 06123 Perugia, Italy

**Keywords:** epithelial–mesenchymal transition (EMT), hepatocellular carcinoma (HCC), TGF-β1 signaling, lipidomic, metabolomic, membrane remodeling

## Abstract

Epithelial–mesenchymal transition (EMT) is a key process in cancer progression and fibrogenesis. In this study, EMT was induced in Huh7 hepatocellular carcinoma cells via TGF-β1 treatment, and the resulting lipidomic and metabolomic alterations were characterized. Morphological changes and protein marker analyses confirmed the transition to a mesenchymal phenotype, with reduced E-cadherin and increased vimentin and N-cadherin expression. Lipidomic profiling revealed a dose-dependent reorganization of membrane lipids, with a pronounced increase in the levels of ceramides, cholesteryl esters, and lysophospholipids, consistent with alterations in membrane structure, potential cellular stress, and modulation of inflammatory pathways. Changes in the content of phospholipid classes, including phosphatidylethanolamines and phosphatidylserines, indicate possible variations in membrane dynamics and potentially point to modifications in mitochondrial function, cellular stress responses, and redox balance. Metabolomic analysis further indicates an alteration of choline and phosphatidylcholine metabolism, consistent with a shift from de novo membrane synthesis toward lipid turnover. Reduced glycolytic capacity and modified acylcarnitine levels indicated impaired metabolic flexibility and mitochondrial efficiency. The integration of phenotypic, lipidomic, and metabolomic data suggests that TGF-β1 induces EMT and drives a coordinated metabolic reprogramming. These findings highlight the involvement of lipid and energy metabolism in sustaining EMT and suggest that specific metabolic reprogramming events characterize the mesenchymal shift in hepatocellular carcinoma. By exploring this process in a tumor-specific context, we aim to deepen our understanding of EMT complexity and its implications for tumor progression and therapeutic vulnerability.

## 1. Introduction

The epithelial–mesenchymal transition (EMT) is a crucial biological process during which epithelial cells, characterized by strong cell–cell junctions and a well-defined apico-basal polarity, acquire a mesenchymal phenotype, characterized by an increased migratory and invasive capacity. This phenotype is associated with loss of cell polarity and disintegration of intercellular junctions [1,2,3]. EMT is vital for physiological processes like tissue regeneration and embryonic development, and it is crucial for tumor progression because it promotes the spread of metastases and increases cellular plasticity [2,4,5]. The suppression of epithelial markers like E-cadherin and the upregulation of mesenchymal markers like N-cadherin, vimentin, and fibronectin are typical indicators of EMT [6,7,8]. A complex network of transcription factors, including Snail, Slug, and Zeb1, regulate these alterations by modifying gene expression, promoting cytoskeletal reorganization, and altering cell adhesive properties [9,10,11]. Furthermore, the process is tightly controlled by regulatory microRNAs, such as the miR-200 family and miR-34, which act in feedback loops with key EMT transcription factors [12,13,14]. Recent research has demonstrated that EMT is a continuum, with intermediate phases known as partial EMT (pEMT), in which cells exhibit both mesenchymal and epithelial traits at the same time [15,16]. This plasticity allows tumor cells to better adapt to microenvironmental changes and optimize their dissemination capacity [5,17].

EMT is not limited to protein changes but also involves metabolic alterations, particularly related to lipid metabolism. A decrease in de novo lipogenesis is acknowledged during EMT, which is supported by a rise in the uptake of exogenous fatty acids, with a preference for polyunsaturated fatty acids (PUFAs) [18,19]. This metabolic remodeling actively promotes tumor growth and increases the resistance to treatment rather than being a passive effect of the phenotypic shift [20]. In particular, key enzymes such as stearoyl-CoA desaturase-1 (SCD1), responsible for the synthesis of monounsaturated fatty acids (MUFAs), and diacylglycerol acyltransferase 1 (DGAT1), involved in the synthesis of triacylglycerols (TAG), are found to be upregulated in the mesenchymal phenotype relative to the epithelial phenotype [21,22,23,24]. Additionally, alterations in the lipid content of membranes, such as the expansion of lipid rafts, affect cell signaling, motility, and tumor cells’ capacity to adapt to harsh environments [25].

Transforming growth factor-beta (TGF-β) is one of the most utilized EMT inducers. This multifunctional cytokine regulates a wide array of cellular processes, including proliferation, differentiation, and apoptosis [26,27,28]. The binding of TGF-β to its receptors activates the SMAD-dependent pathway, wherein phosphorylated SMAD2 and SMAD3 complex with SMAD4 and translocate to the nucleus to regulate the transcription of EMT-related genes. Additionally, TGF-β1 activates SMAD-independent pathways, such as the MAPK, PI3K/AKT, and Rho GTPase pathways, further reinforcing the EMT program [29,30,31]. This molecular reprogramming is driven by EMT-related transcription factors such as Snail, Slug, and Twist, which repress epithelial gene expression and promote mesenchymal gene expression [8,9,10]. These changes culminate in the loss of cell–cell adhesion, reorganization of the cytoskeleton, and increased extracellular matrix production, facilitating cellular migration and invasion. Given its central role in EMT, TGF-β1 is extensively used in vitro to induce EMT and for the study of the phenotypic and molecular changes mechanisms underlying this transition [1,2]. Several studies have used liver cancer cell lines, including HepG2, Hep3B, and SNU-449, to characterize TGF-β1-induced EMT in HCC, showing different degrees of treatment response and variability in the activated molecular programs [19,32,33]. In addition to these, other tumor models, such as the MCF-7 breast cancer cell line, HT-29 colon cancer cell line, and PC3 prostate cancer cell line, have also been used to study TGF-β1-induced EMT, revealing a spectrum of phenotypic and metabolic responses dependent on the cell type and tissue context [34,35,36]. In HCC, TGF-β1-induced EMT is particularly relevant, as it contributes to tumor progression and metastasis [37,38]. HCC is the most common primary liver tumor and a leading cause of cancer-related death worldwide [19,39,40]. Etiological factors such as chronic hepatitis B and C virus infection, excessive alcohol consumption, obesity, and diabetes contribute to its development. EMT is a well-established aspect of HCC progression, promoting local invasion, metastasis, and treatment resistance [39]. Huh7 cells, a human hepatocellular carcinoma-derived cell line, are a widely used experimental model for studying the molecular mechanisms involved in EMT. The well-differentiated epithelial phenotype makes this cell line suitable to study phenotypic and molecular changes following TGF-β1 stimulation, providing a strong system to study EMT-associated alterations [41]. The aim of this work is to investigate metabolic changes related to EMT in Huh7 cells to reveal major changes in this transition, with particular attention to the lipid markers involved. Understanding the interactions between these factors is crucial to identify new therapeutic targets capable of counteracting tumor progression and improving the efficacy of available therapies for hepatocellular carcinoma.

## 2. Materials and Methods

### 2.1. Materials

Phosphate-Buffered Saline (PBS), Dimethyl Sulfoxide (DMSO), and β-actin rabbit monoclonal antibody were purchased from Sigma-Aldrich (St. Louis, MO, USA). Dulbecco’s modified Eagle’s medium (DMEM), fetal bovine serum (FBS), trypsin, and penicillin/streptomycin were purchased from Euroclone (Pero, Italy). The recombinant human TGF-β1 protein, E-cadherin mouse monoclonal antibody, and vimentin XP rabbit monoclonal antibody were purchased from Cell Signaling Technology (Beverly, MA, USA). Huh7 human hepatocellular carcinoma cells were purchased from ATCC (Manassas, VA, USA). EquiSPLASH Lipidomix (Avanti Polar, Alabaster, AL, USA) was purchased for lipidomic analysis. Unless specified otherwise, all other reagents used were of analytical grade and sourced from Sigma-Aldrich (St. Louis, MO, USA).

### 2.2. Cell Culture and EMT Induction

Human HCC cell line Huh7 was maintained at 37 °C in a humidified incubator with 5% CO_2_, using DMEM supplemented with 10% heat-inactivated FBS and antibiotics (100 U/mL penicillin and 100 U/mL streptomycin). For the induction of the epithelial-to-mesenchymal transition process, cells were plated 24 h before treatment, reaching a confluence of 70%. The recombinant human TGF-β1 protein (Cell Signaling Technology, Beverly, MA, USA) was then added to the standard medium in two different concentrations, 10 and 20 ng/mL. The induction and control media were changed every day. The treatment was extended to 72 h. Cell viability was assessed using trypan blue exclusion and quantified with an automated cell counter (Invitrogen™ Countess™, Thermo Fisher Scientific, Waltham, MA, USA) and MTT assay for the evaluation of TGF-β1 cytotoxicity, as reported by Calzoni et al. [42].

### 2.3. Quantitative Real-Time Polymerase Chain Reaction (qRT-PCR)

qRT-PCR was performed to determine the expression levels of EMT-related genes. Total RNA was isolated and extracted from cells using TRIzol RNA Isolation Reagents (Invitrogen, Karlsruhe, Germany).

Then 1 μg of RNA was reversed transcribed into cDNA by using random hexamers and SuperScript II Reverse Transcriptase (Invitrogen) according to the manufacturer’s protocol. Quantitative real-time PCR (RT-PCR) was performed with the primers shown in Table 1. Products were detected with SYBR Green Master Mix (Applied Biosystems, Foster City, CA, USA) using a StepOnePlus thermocycler (Applied Biosystems, Foster City, CA, USA). cDNA abundance was normalized against GAPDH gene. Relative quantification was obtained using the 2−ΔΔCt method.

### 2.4. Immunoblotting

Cells were collected by centrifugation, and the resulting pellets were lysed in RIPA buffer (50 mM Tris-HCl pH 8, 150 mM NaCl, 1% (*v*/*v*) Igepal CA-630, 0.1% (*w*/*v*) SDS, 0.5% (*w*/*v*) sodium deoxycholate) supplemented with a protease inhibitor cocktail (Merck Life Sciences, Darmstadt, Germany). After incubation, cell debris was eliminated by centrifugation at 13,000× *g* for 10 min at 4 °C. Aliquots of cell lysates (30 μg proteins) were mixed with a 5× sample buffer (1 M Tris–HCl pH 6.8, 5% SDS, 6% glycerol, 0.01% bromophenol blue) containing 125 mM DTT and then heated at 95 °C for 5 min to denature the proteins. Samples were then loaded onto a 10% polyacrylamide gel for SDS-PAGE and transferred onto PVDF membranes using a Trans-Blot Turbo Transfer System (Bio-Rad, Hercules, CA, USA). After blocking, membranes were incubated overnight with the following primary antibodies: vimentin mAb, E-cadherin mAb, and N-cadherin mAb (Cell Signaling Technology (Beverly, MA, USA)) and β-actin mAb (Sigma-Aldrich, USA). Detection was performed using HRP-conjugated secondary antibodies (Cell Signaling Technology) according to the manufacturer’s instructions. Immunoreactive bands were visualized via enhanced chemiluminescence (ECL, GE Biosciences, Piscataway, NJ, USA), and densitometric quantification was conducted using ImageJ 1.53 software (NIH, Bethesda, MD, USA).

### 2.5. Fluorescence Microscopy

A total of 1 × 10^3^ cells were plated onto glass coverslips that had been sterilized with 70% ethanol and then placed into a 24-well culture plate (Becton, Dickinson and Company, Franklin Lakes, NJ, USA). After treatment, both induction and control groups were maintained for 72 h in a humidified atmosphere with 5% CO_2_ at 37 °C. Cells were washed with PBS, fixed with 4% paraformaldehyde for 20 min at RT, permeabilized with 0.3% Triton X-100 in PBS/3% FBS for 10 min, and blocked with PBS-3% FBS for 1 h at RT. Samples were then incubated overnight at 4 °C with the anti-human vimentin and E-cadherin antibodies, followed by incubation for 1 h at RT with the Alexa Fluor^®^ 568 secondary antibody. After washing with PBS, samples were mounted with Vectashield^®^ Vibrance™ Antifade Mounting Medium (Vector Laboratories, Newark, CA, USA) containing DAPI to detect nuclei. Fluorescence images were acquired by an Eclipse-TE2000-S (Nikon, Tokyo, Japan) equipped with an F-ViewII FireWire camera (Olympus Soft Imaging Solutions GmbH, Münster, Germany) for images acquisition and processed using CellF Imaging Software (Olympus Soft Imaging Solutions GmbH, Münster, Germany, v 1.4).

The fluorescence intensity of vimentin and E-cadherin in Huh7 was quantified using ImageJ software (version 1.54p). For each cell sample, 5 stained cells were analyzed. Individual cells were selected as regions of interest (ROIs) using the appropriate selection tools. For each ROI, the integrated density, area, and mean gray value were obtained. To account for background fluorescence, five measurements were collected from noncellular areas in each image, and their average intensity was used to calculate the mean background signal. The corrected total cell fluorescence (CTCF) was then calculated according to the established method [48].

### 2.6. Seahorse Glycolytic Activity Analysis

The glycolytic activity of CTRL and EMT-induced cells was analyzed by an Agilent Seahorse XFp Extracellular Flux Analyzer using a Glycolytic Rate assay kit. Cells (2.5 × 10^4^/well) were seeded in XFp cell culture microplates, and after 24 h the medium was replaced with fresh medium with different concentrations of TGF-β1; replacement with fresh medium was performed daily. Upon reaching 72 h of exposure to the cytokine, the medium was replaced with phenol red-free XF DMEM medium supplemented with 10 mM glucose, 2 mM sodium pyruvate, and 2 mM glutamine. To block the electron transport chain and prevent the production of protons derived from CO_2_, mitochondrial respiration was inhibited by adding Rotenone and Antimycin A (Rot/AA), while for the suppression of glycolytic activity, 2-deoxy-D-glucose (2-DG), a glucose analogue, was administered to competitively inhibit hexokinase, the key enzyme initiating glycolysis. The proton efflux rate (PER) is used to show the effects of the induction of the transition process on the glycolytic activity resulting from cellular metabolism.

### 2.7. Lipid and Polar Metabolite Analyses by Liquid Chromatography–Tandem Mass Spectrometry (LC–MS/MS)

For lipid extraction and analysis, aliquots of 1 × 10^6^ cells were processed using the MMC-based method containing Lipidomix SPLASH internal standard, as previously described by Pellegrino et al. [49]. For each cell pellet, 1 mL of a methanol/methyl tert-butyl ether/chloroform mixture (1:1:1, *v*/*v*/*v*) was added. After vortexing, the samples were centrifuged at 16,000× *g* for 10 min at 4 °C. The resulting supernatant was collected, evaporated under a stream of nitrogen at 60 °C, and reconstituted in 100 methanol/toluene solution (9:1, *v*/*v*) for LC–MS analysis.

For analysis of both polar metabolites, aliquots of cells (1 × 10^6^ cells) were prepared for metabolite extraction. The extraction followed the protocol reported by Cajka et al. [50], with slight modifications. Each cell pellet was first mixed with 275 µL of methanol containing the Lipidomix SPLASH internal standard and vortexed for 30 s. Then, 750 μL of methyl tert-butyl ether (MTBE) was added, and the samples were vortexed again, followed by 20 min of shaking at 1500 rpm on a T-Shaker (Euroclone). Next, 188 μL of water was added for the samples. The samples were centrifuged at 16,000× *g* for 10 min at 4 °C to achieve phase separation.

The lower (aqueous) phase was recovered and dried under a nitrogen steam at 60 °C. The resulting dry extract was reconstituted in 100 µL of a 4:1 acetonitrile/water solution and placed in an autosampler for polar metabolites analysis.

Chromatographic separation and data acquisition was performed using an UHPLC-QTOF system (Agilent 1260 Infinity II liquid chromatograph coupled with an Agilent 6530 Accurate-Mass Q-TOF mass spectrometer equipped with a JetStream ESI source, Agilent Technologies, Santa Clara, CA, USA). The LC-MS settings for both lipid and polar metabolite analyses followed the protocol described by Alabed et al. [51].

### 2.8. Statistical Analysis

Statistical analyses and graph generation for growth curves, gene expression, densitometric quantification, and log CTFC fluorescence intensity were performed using GraphPad Prism (version 9.1). Data were analyzed by one-way ANOVA followed by Tukey’s post hoc test for multiple comparisons. Each experiment was conducted with three independent biological replicates.

Lipidomic profiling was analyzed using the open-source tool LipidOne 2.3 [52], with five biological replicates per treatment group. Data preprocessing included median centering, log transformation, and autoscaling followed by statistical analysis with one-way ANOVA and Tukey’s post hoc test. Principal Component Analysis (PCA) was used as an unsupervised method to explore sample distribution. Hierarchical clustering heatmaps were generated to visualize patterns of lipid expression across samples. Partial Least Squares Discriminant Analysis (PLS-DA) was applied, and the resulting VIP (Variable Importance in Projection) scores were used to rank the top 20 discriminant lipids, visualized alongside their group-specific expression profiles. Lipid pathway and gene involvement analysis was conducted using LipidOne version 2.3 to annotate lipidomic alterations based on specific biochemical pathways using the STRING database (Homo sapiens). The Gene Involvement function associates lipid species with enzymatic reactions and infers potential changes in enzymatic activity through statistical comparisons between experimental groups. For each reaction, a t-score is calculated that reflects both the direction and magnitude of changes in lipid levels associated with that enzymatic step: positive t-scores indicate potential upregulation, while negative values suggest downregulation.

Metabolomic data were processed and analyzed using MetaboAnalyst (version 5.0), applying the same normalization and statistical procedures as described above. PCA, heatmap and pathway analysis were performed using default settings. The analysis combined quantitative metabolite data with pathway information from the KEGG database (Homo sapiens). Statistical enrichment was based on hypergeometric testing, and pathway impact was calculated using relative betweenness centrality.

## 3. Results

### 3.1. EMT In Vitro Model Induction and Characterization

**Figure 1:**
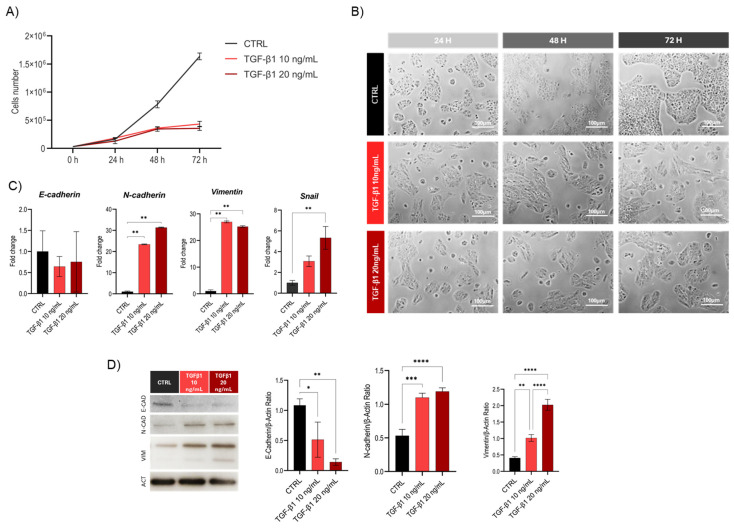
Analysis of EMT induction in the Huh7 cell line. (**A**) Cell proliferation assay with TGF-β1 at 10 and 20 ng/mL compared to untreated control (CTRL) over 72 h. Data are expressed as mean ± SD (*n* = 3). (**B**) Phase-contrast microscopy images for morphological analysis. Scale bar = 100 µm. (**C**) Quantitative RT-PCR analysis of mesenchymal markers N-cadherin, vimentin, and Snail following TGF-β exposure. Data are shown as fold change relative to control, and GAPDH was used for normalization (mean ± SD, *n* = 3). (**D**) Western blot analysis and quantification of protein markers. β-actin was used as a loading control. Quantification (right) shows the E-cadherin/β-actin, N-cadherin/β-actin and vimentin/β-actin ratios (mean ± SD, *n* = 3). Statistical analysis for gene (**C**) and protein (**D**) expression: * *p* < 0.05, ** *p* < 0.01, *** *p* < 0.001, **** *p* < 0.0001 (one-way ANOVA followed by Tukey’s post hoc test).

To induce the EMT process, Huh7 cells at 70% confluence were treated with two different concentrations of TGF-β1 (10 and 20 ng/mL) for 72 h. Prior to selecting these concentrations, a preliminary MTT assay was performed to assess cell viability across a range of TGF-β1 concentrations (2.5–30 ng/mL), confirming the absence of cytotoxic effects after 24 and 48 h of treatment (Figure S1). To evaluate the EMT model, growth curves and changes in cell morphology, as well as the expression of key gene and protein markers, were assessed (Figure 1).

Cells were treated with 10 ng/mL and 20 ng/mL TGF-β1 for 72 h. Following treatment, a clear reduction in cell proliferation was observed starting at 48 h of TGF-β1 exposure compared to the control for both concentrations, as shown in Figure 1A. However, no significant differences were detected between the two treatment concentrations, suggesting that the reduction in cell proliferation does not follow a dose-dependent trend within this range. This data is also strengthened by the microscopic analyses of cell morphology; in fact, as shown in Figure 1B, treated cells exhibit an altered structure, appearing more elongated and less compact compared to controls. These morphological changes are consistent with the acquisition of a mesenchymal phenotype, which appears to correlate with both duration and concentration of the inducing cytokine exposure.

The analysis of gene expression of EMT markers was performed by quantitative real-time PCR using the relative quantification method (ΔΔCt), with normalization to the housekeeping gene GAPDH. The results revealed that TGF-β1 induces an increase in the transcription of key mesenchymal proteins, such as vimentin and N-cadherin (Figure 1C). Additionally, in the case of the 20 ng/mL treatment condition, a significant increase in the transcriptional factor Snail was also observed (Figure 1C). In contrast, the decrease in the E-cadherin gene transcription does not appear significant at the transcriptional level. However, protein expression analysis (Figure 1D) showed a decrease in E-cadherin levels, suggesting that its regulation may occur primarily at the post-translational level. This is supported by the literature evidence showing that miR-9 and miR-23a directly bind to E-cadherin mRNA, inhibiting its expression and promoting cell migration and invasiveness [53,54]. The increase in vimentin and N-cadherin protein expression confirmed the transcriptional data, reinforcing the mesenchymal shift induced by the treatment.

**Figure 2:**
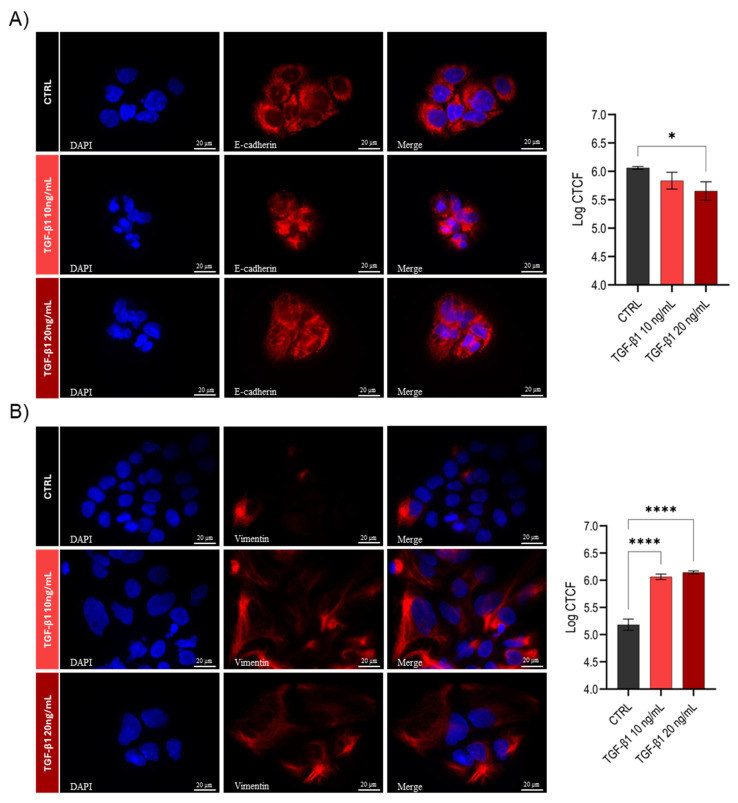
Immunohistochemical images of the Huh7 EMT model. E-cadherin (**A**) and vimentin protein (**B**) expression in CTRL and treated cells in EMT model. Quantification of fluorescence intensity was performed using log-transformed corrected total cell fluorescence (log CTCF) values (mean ± SD, *n* = 3). Statistical analysis was conducted using one-way ANOVA followed by post hoc testing. Significance is indicated as follows: * *p* < 0.05; **** *p* < 0.0001.

To strengthen these results, an immunohistochemical analysis was additionally carried out (Figure 2).

The immunohistochemical images of the transition model highlighted the intracellular localization of the analyzed markers and revealed morphological changes associated with the transition process. In particular, E-cadherin signal intensity showed a significant reduction (*p* < 0.05) at the highest TGF-β1 concentration. Additionally, in the TGF-β1-treated cells, its subcellular localization appeared more diffuse and less distinctly membrane-associated compared to controls (Figure 2A). As for vimentin, the images confirm the significant increase observed at both the gene and protein levels (Figure 1C,D), further highlighting how the cytoskeletal structure of the cell appears more elongated and branched, typical of a mesenchymal phenotype (Figure 2B).

### 3.2. Lipidomic Profile of EMT Model

Given the central role of lipid metabolism in cancer progression and EMT [55], the lipidomic profile of Huh7 cells undergoing TGF-β-induced EMT was analyzed to identify potential lipid-based markers associated with this phenotypic transition using an LC/MS approach. A total of 183 lipid species, belonging to 18 different lipid classes and subclasses, were annotated (Table 2).

Lipid analysis revealed noteworthy alterations in the lipidomic profile induced by TGF-β1 treatment, particularly at the higher concentrations. Among the significantly modulated classes, cholesteryl esters (CE) showed a marked dose-dependent increase, with pairwise comparisons indicating significant differences between CTRL and TGF-β1 20 ng/mL, as well as between TGF-β1 10 ng/mL and 20 ng/mL (Table 2). This may reflect changes in sterol metabolism and increased cholesterol esterification, a process linked to protection from lipotoxicity, energy storage, and tumor progression through modulation of oncogenic pathways [56,57]. The accumulation of ceramides (Cer), a known mediator of cellular stress, apoptosis, and inflammatory responses, and hexosyl-ceramides (HexCer) indicates that TGF-β1 may induce a pro-apoptotic or inflammatory state of cellular activation. This is further supported by Tukey’s HSD analysis, which revealed significant differences across all condition pairs for both lipid classes (Table 2), highlighting a consistent and progressive accumulation in response to increasing TGF-β1 concentrations. Some evidence in the literature has associated a decrease in ceramides with EMT, while increased ceramide levels have been observed in HCC patients, particularly those with long acyl chains, suggesting a tumor-specific lipid remodeling related to metabolic organism state and systemic liver function [58,59,60]. Moreover, Piacentino et al. demonstrated that during embryonic development, ceramide production is upregulated at the onset of EMT in neural crest cells, thereby promoting pro-migratory gene expression and facilitating EMT. These findings highlight the crucial role of plasma membrane lipid metabolism in the transcriptional regulation of EMT albeit in a non-pathological context [61]. In parallel, the increase in the two species included in the classes of lysophospholipids (LPE and LPI) may suggest enhanced membrane phospholipid turnover, likely reflecting active remodeling processes, such as those mediated by phospholipase A2 activity within the Lands cycle [62,63,64]. Moreover, a significant decrease in phosphatidic acid (PA) with significant differences between CTRL and both TGF-β1 concentrations as well as an increase in phosphatidylethanolamines (PE) and their ether and vinyl derivatives (PE-O and PE-P) were also observed in particular in the 20 ng/mL concentration (Table 2). In addition, modulation at the levels of phosphatidylserines (PS) and phosphatidylglycerols (PG) was detected and is reported in Table 2; this showed significant differences between CTRL and both TGF-β1 treatments for PS and between CTRL and TGF-β1 20 ng/mL for PG, as well as between the two TGF-β1 doses. These lipid classes are primarily associated with internal cellular membranes in the case of PG, whereas PS are also enriched in the inner leaflet of the plasma membrane, where they play key roles in membrane asymmetry and signaling [65,66]. A list of all annotated lipid molecular species, including their semi-quantification and statistical significance across conditions, is provided in Table S1, offering a detailed overview of the lipidomic changes induced by TGF-β1 treatment.

In panels D and E, the plot displays t-scores associated with significant changes in lipid levels, with blue bars indicating increases and red bars indicating decreases. The height of each bar represents the magnitude of the change. Analyses were performed on lipidomic data obtained from CTRL and TGF-β1-treated Huh7 cells. Data were normalized by median centering, log-transformed, and autoscaled before multivariate analysis using LipidOne 2.3.

**Figure 3:**
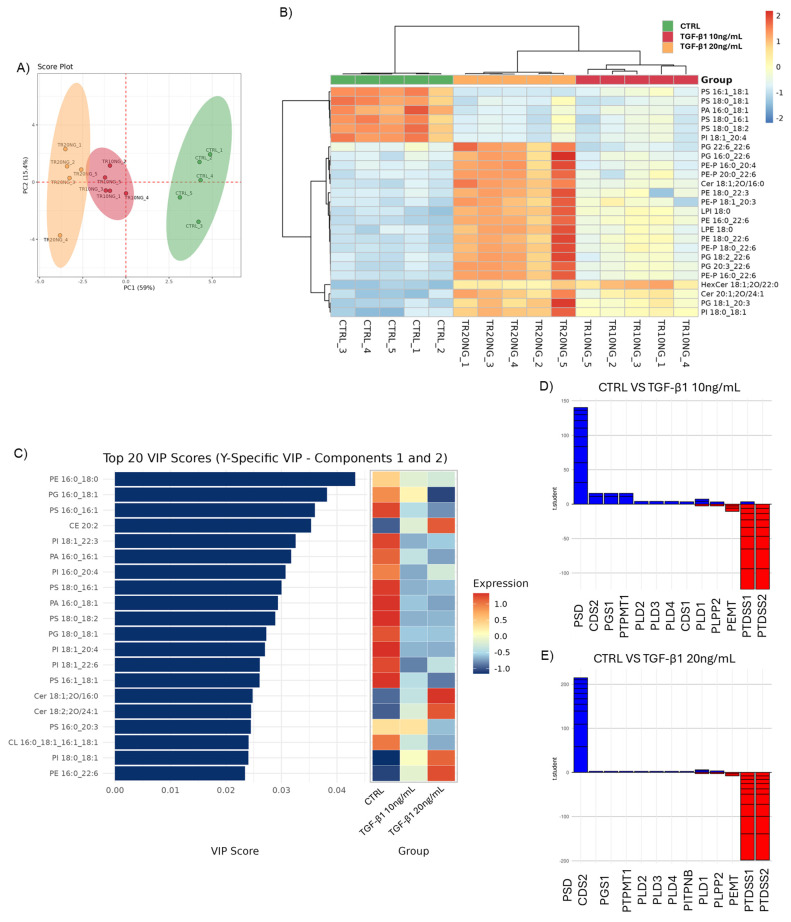
Lipidomic analysis of Huh7-induced EMT. Principal Component Analysis (PCA) score plot (**A**), hierarchical clustering heatmap (**B**), Variable Importance in Projection (VIP) scores from PLS-DA with corresponding expression heatmap (**C**), gene involvement plot showing t-scores of significant lipid transformations, comparing CTRL vs. TGF-β1 10 ng/mL (**D**) and CTRL vs. TGF-β1 20 ng/mL (**E**). In (**B**), the color scale represents the relative lipid abundance (log2 fold change), ranging from blue (lower expression) to red (higher expression). In (**C**), the heatmap shows lipid expression levels across groups, with colors from blue (low) to red (high) expression.

As shown in Figure 3A, treatments cluster perfectly in the Principal Component Analysis (PCA), clearly separating the control from the treated groups along the x-axis with a noteworthy variance of 59%. Furthermore, it can also be noted that although there is a partial overlap between the two treatments, the group treated with a higher concentration of TGF-β1 (20 ng/mL) shifts further away from the control, supporting the hypothesis of a dose-dependent EMT induction. In support of the above analysis, it can also be observed that in Figure 3B, the heatmap of the 25 most significant lipid molecular species reveals clear clustering of the samples according to treatment groups, highlighting several lipid species that could potentially play a key role in the transition process.

Furthermore, the analysis of VIP scores (Variable Importance in Projection) (Figure 3C) further identifies the most discriminating lipids between the experimental groups. Among the lipid species with high VIP scores are phosphatidylethanolamines (PE 16:0_18:0), phosphatidylglycerols (PG 16:0_18:1), phosphatidylserines (PS 16:0_16:1), and phosphatidylinositols (PI 18:1_22:3) (lipid class does not exhibit significant changes at the bulk level; however, several constituent molecular species are significantly modulated), suggesting a significant remodeling of cell membranes. The associated heatmap (Figure 3B) shows that the abundance of several of these lipids varies gradually across treatment conditions, indicating a dose-dependent modulation.

Based on the observed changes in the lipid profile, an in silico analysis of the associated biosynthetic pathways was performed (Figure 3D,E) using LipidOne 2.3, aiming to identify potential transcriptional and/or enzymatic regulation consistent with the variations in lipid classes in both treatment conditions. The decrease in PS, PA, and some members of PI suggests a repression of the biosynthetic pathways responsible for their direct synthesis. In line with this trend, comparison with known pathways indicates a putative decrease in the enzymes PTDSS1 and PTDSS2, which synthesize phosphatidylserine via base-exchange reactions using phosphatidylcholine and phosphatidylethanolamine, respectively [67]. In parallel, a possible increase in CDS2, PGS1, and PSD, known to be involved in the synthesis of key intermediates such as CDP-DAG, PG, and PE from PS, is observed, suggesting a redirection of lipid flux towards alternative routes or a compensatory response. This profile indicates a remodeling of glycerophospholipid biosynthesis, with potential implications for membrane composition, lipid-mediated signaling, and organellar dynamics.

### 3.3. Metabolic Profile of the EMT Model

**Figure 4:**
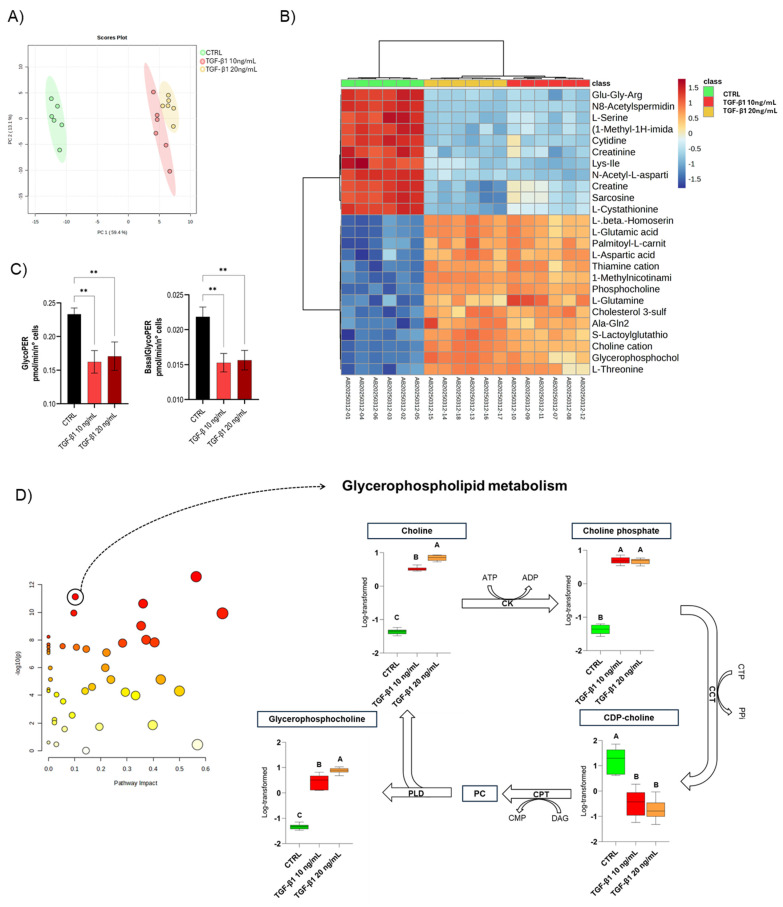
Metabolomic analysis of Huh7-induced EMT. Principal Component Analysis (PCA) score plot (**A**), hierarchical clustering heatmap (**B**), bar graphs of glycolytic (GlycoPER) and basal mitochondrial (BasalGlycoPER) (**C**) asterisks indicate the significance level of the overall ANOVA: ** *p* < 0.01 pathway analysis and boxplots displaying normalized levels of key choline-related metabolites (**D**). Abbreviations: CK, choline kinase; CCT, CTP:phosphocholine cytidylyltransferase; CTP, cytidine triphosphate; CPT, CDP-choline:1,2-diacylglycerol cholinephosphotransferase; PC, phosphatidylcholine; PLD, phospholipase D; CDP-choline, cytidine diphosphate-choline; ATP, adenosine triphosphate; ADP, adenosine diphosphate; PPi, inorganic pyrophosphate; DAG, diacylglycerol; CMP, cytidine monophosphate; *p* < 0.05 by one-way ANOVA or appropriate test as indicated. Data were normalized by median centering, log-transformed, and autoscaled before multivariate analysis. Boxplots represent median, interquartile range (IQR), and individual sample values. Different letters above boxplots indicate statistically significant differences based on post hoc multiple comparison tests.

An untargeted metabolomic analysis was also performed to complete the analytical framework of the in vitro model, which highlighted a significant reorganization of the cellular metabolic profile in response to TGF-β1 treatment that involved 122 annotated compounds, which were characterized through MetaboAnalyst (Figure 4).

The clusterization between the control and treated groups observed in the PCA (Figure 4A) showed a remarkable alteration of metabolism, with 59.4% of the total variance explained by the first principal component. This separation was further corroborated by the hierarchical clustering analysis of the heatmap (Figure 4B), which revealed well-defined and consistent metabolic patterns among the treated samples, confirming the robustness of the TGF-β1 treatment and its dose-dependent response. The heatmap highlights several metabolic classes, including amino acids and their derivatives, metabolites involved in choline and phospholipid metabolism, energy-related compounds such as creatine and creatinine, intermediates of nucleotide metabolism, and molecules associated with the oxidative stress response and osmotic balance, reflecting a broad metabolic reorganization in response to TGF-β1 treatment.

In particular, metabolites involved in choline and phospholipid metabolism were significantly altered. Increased levels of choline, phosphocholine, and glycerophosphocholine were observed in treated samples, particularly at the higher concentration (20 ng/mL), as shown in the heatmap. This pattern again suggests active membrane phospholipid remodeling, consistent with structural adaptations characteristic of the fibrotic phenotype. Choline metabolism plays a pivotal role in membrane biogenesis, signal transduction, and lipid homeostasis. The accumulation of choline derivatives suggests increased demand for membrane synthesis and reorganization, likely reflecting a shift in cellular architecture required for mesenchymal transition. It was demonstrated that choline kinase alpha (CHKα), an enzyme catalyzing the phosphorylation of choline, was upregulated in both tumorigenic and fibrotic EMT contexts, contributing to enhanced migratory potential and TGF-β pathway activation [65,66]. Moreover, phospholipid intermediates such as phosphatidylcholine are known to modulate intracellular signaling cascades, including PI3K/Akt and MAPK, which are essential for EMT induction and maintenance [68,69,70]. These results are consistent with previous observations indicating that glycerophospholipid metabolism plays a crucial role in the cellular response to fibrotic stimuli [71,72].

Analysis of energy metabolism using a Seahorse Glycolytic Rate Assay (Figure 4C) showed a reduction in glycolytic rate (GlycoPER) in TGF-β1-treated cells compared to control, suggesting a limited capacity to activate glycolysis as a compensatory pathway for energy production. This finding, together with the absence of an increased glycolytic response after mitochondrial inhibition, suggests an impairment of metabolic flexibility and a potential dysfunction of intracellular bioenergetic mechanisms. In particular, lipidomic data highlight alterations in lipid classes associated with mitochondrial functionality, including PG and cardiolipins (CL) (in which only two species are found to be significantly modulated by the treatment; Table S1), essential for the stability and activity of electron transport chain complexes. Their variation is often indicative of alterations in oxidative phosphorylation and the structure of the inner mitochondrial membrane. Furthermore, the presence of changes in acylcarnitine levels, including a significant accumulation of palmitoylcarnitine, may indicate a reduced efficiency in fatty acid β-oxidation, a crucial process that occurs within the mitochondria. Additional increases in related metabolites such as acetyl-DL-carnitine, butyrylcarnitine, and propionylcarnitine further support the involvement of impaired mitochondrial fatty acid metabolism (Supporting Table S2). These findings can be consistent with the occurrence of treated cells duplicating more slowly and their energy requirement perhaps being limited. This interpretation is consistent with studies showing that TGF-β1 signaling can impair β-oxidation by downregulating mitochondrial enzymes such as CPT1 in hepatocytes and fibrotic models [73]. Conversely, other reports indicate that TGF-β1 can enhance fatty acid oxidation in certain cancer or EMT contexts to sustain energy production and cell migration, highlighting the context dependence of its metabolic effects [19,74]. Simultaneous alterations of Cer and lysophospholipids, known effects of oxidative stress and membrane damage, strengthen the hypothesis of an intracellular environment with increased ROS production [75].

To better understand how TGF-β1 affects cellular metabolism, we used MetaboAnalyst to perform pathway analysis. This method combines the enrichment analysis to identify pathways with a significant number of altered metabolites based on *p*-values and pathway topology analysis to assess how central those metabolites are within each pathway, generating an impact score. Using default settings, the analysis revealed a significant impact on the glycerophospholipid pathway, with very low *p*-values and a high impact on the pathway, suggesting a critical role of choline metabolism in the response to TGF-β1 (Figure 4D).

Specifically, boxplots of key choline-related metabolites show a significant accumulation of choline, phosphocholine, and glycerophosphocholine, associated with a marked decrease in CDP-choline. This pattern indicates a metabolic bottleneck at the level of CTP:phosphocholine cytidylyltransferase (CCT), the enzyme responsible for converting phosphocholine into CDP-choline.

Furthermore, the observed increase in free choline and glycerophosphocholine may indicate enhanced PC catabolism, possibly via phospholipase D (PLD) activity, as suggested in the gene involvement plots in Figure 3D,E. This enzymatic remodeling suggests a shift from de novo membrane synthesis toward PC turnover, potentially supporting rapid membrane reorganization or signaling lipid generation. Upstream, choline kinase (CK) appears to remain active, as evidenced by elevated phosphocholine levels, indicating that early steps of the Kennedy pathway are preserved or upregulated. The final step of PC biosynthesis, catalyzed by CDP-choline:1,2-diacylglycerol cholinephosphotransferase (CPT), may also be limited due to substrate (CDP-choline) depletion.

These metabolic changes are aligned with the lipidomic data, which reveal a selective depletion of specific phosphatidylcholine molecular species in response to TGF-β1 stimulation. Although the overall abundance of the PC class was not significantly affected, a focused analysis identified four PC species that were significantly reduced upon TGF-β1 treatment: PC 16:0_18:1, PC 14:0_14:0, PC 16:0_16:0, and PC 14:0_16:0 (Figure S2). These reductions were dose-dependent and most pronounced at 20 ng/mL of TGF-β1, suggesting a tightly regulated, species-specific turnover of membrane phospholipids. The downregulation of both saturated and monounsaturated PCs may reflect a remodeling of membrane fluidity consistent with dynamic cellular reshaping processes. This pattern, together with the accumulation of choline, phosphocholine, and glycerophosphocholine and the depletion of CDP-choline, suggests a TGF-β1-driven metabolic reprogramming of the choline–phospholipid axis.

## 4. Discussion

The present study provides a comprehensive characterization of the lipidomic and metabolomic alterations induced by TGF-β1 stimulation in Huh7 cancer cells. The data demonstrate that TGF-β1 exerts a multifaceted impact, not only promoting epithelial–mesenchymal transition (EMT) and fibrogenesis but also inducing an important metabolic reprogramming, particularly involving lipid and energy metabolism. Morphological analysis confirmed the induction of EMT, as evidenced by a clear change in cellular organization, with loss of epithelial morphology, upregulation of mesenchymal markers such as vimentin, and downregulation of E-cadherin. This effect is accompanied by a reduction in cell proliferation and an increase in N-cadherin protein expression, indicating an activation towards a fibroblastic phenotype.

In addition, lipidomic analysis also revealed significant changes in lipid species typically associated with cellular membranes, suggesting active membrane remodeling in response to TGF-β1-induced EMT, particularly a significant accumulation of ceramides and lysophospholipids, which are known to be associated with cellular stress, membrane damage, and pro-apoptotic signaling [19,76]. In particular, the increase in ceramides suggests the activation of pro-apoptotic or inflammatory pathways [76,77,78]. Although previous studies have reported decreased ceramide levels in HCC [59], often associated with reduced apoptosis and increased tumor proliferation, our results demonstrate an increase in ceramides following TGF-β1 stimulation in Huh7 cells. This discrepancy may be explained by the specific cellular environment and the phase of tumor progression in vitro, in which TGF-β1 induces significant stress and metabolic reprogramming. These conditions could promote the transient accumulation of ceramides as a pro-apoptotic or pro-inflammatory signal. Furthermore, it could be a transient stimulus that activates the expression of genes associated with EMT and cell motility [61]. This suggests that ceramide levels reflect a dynamic balance between synthesis and degradation, influenced by microenvironmental factors and signaling. This supports the idea that ceramides not only function as mediators of apoptosis but also participate in the cellular phenotypic transitions characteristic of TGF-β1-induced HCC progression.

Furthermore, the observed modulation of phosphatidic acids, phosphatidylethanolamines, and their ether (PE-O) and vinyl ether (PE-P) derivatives may reflect cellular adaptation to oxidative or mitochondrial stress. These lipids are integral components of inner mitochondrial and endoplasmic reticulum membranes, where they contribute to curvature, membrane fusion, and regulation of organelle dynamics under stress conditions [79,80]. Similarly, the decrease in phosphatidylserines and increase in phosphatidylglycerols may be linked to alterations in mitochondrial membrane composition and homeostasis. In particular, PS externalization is a well-established hallmark of early apoptosis, while PG is involved in cardiolipin biosynthesis, further connecting these changes to mitochondrial integrity and potential activation of apoptotic signaling pathways [80]. Although cardiolipins did not show a statistically significant change in abundance, a decreasing trend was noted in treated samples. This observation is particularly relevant given the key role of cardiolipins in stabilizing the inner mitochondrial membrane and supporting respiratory chain function [81,82,83]. These findings are in line with previous lipidomic and metabolic studies conducted in other cancer cell models undergoing EMT. For example, in A549 lung cancer cells, EMT induction by TGF-β1 has been associated with a significant accumulation of ceramides and lysophosphatidylcholines, mirroring the lipid profile observed in our Huh7 cells [84].

Similarly, in breast cancer models such as MDA-MB-231 and MCF7, EMT transition has been reported to trigger major shifts in phospholipid metabolism, including changes in phosphatidylethanolamine and phosphatidylcholine species, often linked to mitochondrial dynamics and increased membrane fluidity [85,86]. Among the top VIP-ranked lipids, several mitochondrial-associated phospholipids were identified, including PG and cardiolipins, supporting the hypothesis of structural remodeling of internal membranes. PE and PS are essential for maintaining mitochondrial architecture and bioenergetic capacity and are also implicated in apoptotic regulation [87,88]. The increased representation of phosphatidylinositols (PI), particularly PI 18:1_22:3 and PI 16:0_20:4, may reflect changes in phosphoinositide signaling cascades, potentially involving TGF-β1-responsive pathways such as PI3K/AKT [89,90]. The enrichment of PA further supports the presence of active membrane biogenesis and remodeling. Ceramides, through their roles in inflammation and apoptosis, strengthen the interpretation of a stress-related lipidomic signature. Finally, the inclusion of cholesteryl ester CE 18:2 among the most discriminative species suggests possible alterations in cholesterol metabolism or storage, with implications for membrane homeostasis and lipid droplet dynamics [57]. This increase has been widely demonstrated in fibrotic and metastatic processes such as pancreatic cancer [56]. Such changes may reflect altered sterol metabolism and increased cholesterol esterification, a process linked to protection from lipotoxicity, energy storage, and tumor progression through modulation of oncogenic pathways [56,57]. Overall, the Variable Importance in Projection (VIP) scores highlight the central role of mitochondrial- and membrane-associated lipid classes in driving the observed lipidomic differences between treatment conditions [91,92].

In support of the hypothesis of mitochondrial involvement, metabolomic data show alterations in the levels of metabolites involved in choline and phospholipid metabolism (phosphocholine, CDP-choline, sn-Glycero-3-phosphocholine), indirect markers of membrane turnover. Pathway impact analysis identified the choline biosynthesis and glycerophospholipid metabolism pathways as among the most affected, reinforcing the hypothesis of structural and functional membrane remodeling.

In addition, the analysis of energy metabolism by Seahorse XF Glycolytic Rate Assay reveals a reduction in the glycolytic rate (GlycoPER) in TGF-β1 treatments. This indicates a compromised glycolytic capacity of the cells and a potential metabolic rigidity. The absence of a glycolytic response following mitochondrial inhibition suggests that the treated cells have lost the capacity to shift their energy production strategy in response to metabolic stress. These findings are consistent with the observed lipid alterations, particularly the accumulation of ceramides, changes in cardiolipin levels, and reduction in acylcarnitines, which are indicative of mitochondrial stress, impaired β-oxidation, and altered oxidative phosphorylation [93,94]. Moreover, metabolic reprogramming during EMT has been shown to involve the suppression of glycolytic flux and upregulation of oxidative metabolism also in colorectal and pancreatic cancer cell lines [95,96], consistent with our observation of reduced glycolytic activity in Huh7 cells following TGF-β1 exposure.

In line with these findings, a focused analysis of phosphatidylcholine species revealed a selective depletion of short-chain and saturated PCs despite no overall quantitative change in total PC levels. This suggests a fine-tuned remodeling of membrane composition, likely driven by altered enzymatic flux within the Kennedy pathway. These results further support the hypothesis that TGF-β1 treatment induced perturbation of glycerophospholipid metabolism, contributing to structural membrane reorganization and bioenergetic adaptation [97,98]. Although the tumor-specific nature of EMT imposes certain limitations, these findings provide valuable insights into the metabolic and lipidomic reprogramming associated with EMT in hepatocellular carcinoma, enhancing the understanding of its biological and pathological significance.

## 5. Conclusions

The analysis of phenotype, lipidomic profile, and cellular metabolism of EMT-induced Huh7 allowed us to delineate in detail the effects of TGF-β1 treatment in hepatocellular carcinoma cells. The obtained results demonstrate that TGF-β1 stimulation promotes the activation of the epithelial–mesenchymal transition process in association with an important remodeling of lipid and energy metabolism. In particular, a consistent reorganization of membrane lipid composition emerged, with an accumulation of bioactive species associated with oxidative stress, apoptosis, and mitochondrial dysfunction. These results are corroborated by a marked alteration of metabolic pathways related to choline metabolism, phospholipids, and cell membrane turnover, as well as by a reduced metabolic flexibility evidenced by the impairment of the glycolytic response. The set of these alterations supports the hypothesis of a functional and structural reconfiguration of the cell in response to fibrogenic stimuli, suggesting that TGF-β1 acts as a key regulator of a pro-fibrotic and mitochondria-dependent metabolic setup. Such metabolic adaptations could represent relevant therapeutic targets in pathological contexts characterized by EMT or tumor progression.

## Figures and Tables

**Table 1:** Primer sequences.

Sequence 5′-3′	Name	Reference
TGCACCACCAACTGCTTAGC	GAPDH F	[43]
GGCATGGACTGTGGTCATGAG	GAPDH R	
AGCCTCAGGTCATAAACATCATTG	E-cadherin F	[44]
GATAGATTCTTGGGTTGGGTCG	E-cadherin R	
ATTGGACCATCACTCGGCTTA	N-cadherin F	[45]
CACACTGGCAAACCTTCACG	N-cadherin R	
AGTCCACTGAGTACCGGAGAC	Vimentin F	[46]
CATTTCACGCATCTGGCGTTC	Vimentin R	
TCGGAAGCCTAACTACAGCGA	Snail F	[47]
AGATGAGCATTGGCAGCGAG	Snail R	

**Table 2:** Lipid classes semi-quantified in CTRL, TGF-β1 10ng/mL, and TGF-β1 20 ng/mL (mean ± SE, *n* = 5). Statistical analysis was performed using one-way ANOVA followed by Tukey’s post hoc test for multiple pairwise comparisons. The *p*-values reported in the table refer to the ANOVA test. Different lowercase letters (a, b, c) indicate statistically significant differences between groups (CTRL; TGF-β1 10 ng/mL and 20 ng/mL) in the same lipid class. Asterisks indicate the significance level of the overall ANOVA: * *p* < 0.05, ** *p* < 0.01, *** *p* < 0.001.

Class	Count	CTRL		TGF-β1 10 ng/mL		TGF-β1 20 ng/mL		*p*-Value	Sign.
CE	5	2.53173 ± 0.15611	b	3.22515 ± 0.40402	b	5.49683 ± 0.41794	a	1.54 × 10^−4^	***
CL	9	0.10918 ± 0.01037		0.08659 ± 0.00393		0.09885 ± 0.00695		1.49 × 10^−1^	
Cer	12	0.02440 ± 0.00110	c	0.03244 ± 0.00122	b	0.04826 ± 0.00152	a	6.49 × 10^−8^	***
DG	9	1.00271 ± 0.03512		1.31249 ± 0.17875		1.56231 ± 0.20878		8.38 × 10^−2^	
HexCer	3	0.00230 ± 0.00006	c	0.00408 ± 0.00021	a	0.00338 ± 0.00014	b	8.47 × 10^−6^	***
LPC	1	0.02879 ± 0.00059	a	0.01318 ± 0.00319	b	0.02019 ± 0.00466	a	1.83 × 10^−2^	*
LPE	1	0.00992 ± 0.00053	c	0.01316 ± 0.00063	b	0.02142 ± 0.00087	a	1.90 × 10^−7^	***
LPI	1	0.00339 ± 0.00021	c	0.00538 ± 0.00021	b	0.00838 ± 0.00035	a	6.37 × 10^−8^	***
PA	3	0.05569 ± 0.00248	a	0.02675 ± 0.00146	b	0.02398 ± 0.00247	b	3.86 × 10^−7^	***
PC	23	6.93216 ± 1.30989		4.72462 ± 0.74767		5.72477 ± 1.31097		4.26 × 10^−1^	
PE	24	1.65486 ± 0.05482	b	1.89319 ± 0.14558	b	2.61706 ± 0.16727	a	6.40 × 10^−4^	***
PE-O	2	0.00474 ± 0.00020	c	0.00648 ± 0.00051	b	0.00869 ± 0.00053	a	1.36 × 10^−4^	***
PE-P	5	0.00957 ± 0.00026	c	0.01337 ± 0.00080	b	0.02592 ± 0.00124	a	3.57 × 10^−8^	***
PG	12	0.13965 ± 0.00445	b	0.14419 ± 0.00732	b	0.18684 ± 0.01239	a	4.16 × 10^−3^	**
PI	18	2.20432 ± 0.06209		2.01694 ± 0.08885		2.31428 ± 0.10065		8.22 × 10^−2^	
PS	13	1.92422 ± 0.05494	a	1.45601 ± 0.06127	b	1.59096 ± 0.08233	b	1.04 × 10^−3^	**
SM	13	1.39350 ± 0.30270		1.15554 ± 0.22155		1.41455 ± 0.35496		7.96 × 10^−1^	
TG	29	7.63852 ± 0.77395		13.54987 ± 2.91798		19.20528 ± 8.68190		3.38 × 10^−1^	
Total	183	25.66967 ± 1.95333		29.67943 ± 3.13796		40.37196 ± 8.40289		1.71 × 10^−1^	

## Data Availability

Dataset available on request from the authors.

## Supplementary Materials

The following supporting information can be downloaded at: https://www.mdpi.com/article/10.3390/cells14161233/s1, Figure S1: Cell viability assessed by MTT assay at different concentrations of TGF-β. Results are expressed as percentage relative to untreated control cells (CTRL); Figure S2: The figure shows four boxplots representing the concentrations (in µg/10⁶ cells) of different phosphatidylcholine (PC) species: PC 16:0_18:1 (A); PC 14:0_14:0 (B); PC 16:0_16:0 (C); PC 14:0_16:0 (D). Each boxplot shows the distribution of values with the median (center line), quartiles (boundaries of the box), and outliers (outer dots). ** indicates a statistically significant difference with a p-value < 0.01; * indicates a statistically significant difference with a *p*-value < 0.05; Table S1: The table reports the mean ± SD (N = 5) of each lipid species quantified in the lipidomic dataset across three experimental conditions: CTRL, TGF-β1 10 ng/mL, and TGF-β1 20 ng/mL. Lipid abundances were normalized and compared using one-way ANOVA followed by Tukey’s HSD post hoc test. For each species, unadjusted and FDR-adjusted p-values are provided. Results of multiple comparisons between experimental conditions (denoted as A, B and C respectively CTRL, TGF-β 10 ng/mL, and TGF-β 20 ng/mL) using Tukey’s HSD test; Table S2: The table reports the mean ± SE (N = 6) of the normalized peak areas (median centering, log transformation, and autoscaling) of metabolic LC/MS dataset. For each variable the compared condition pairs, unadjusted p-values, and FDR-adjusted p-values are reported. Statistically significant differences (FDR < 0.05) indicate lipid alterations associated with TGF-β treatment. Results of multiple comparisons between experimental conditions (denoted as A, B and C respectively CTRL, TGF-β 10 ng/mL, and TGF-β 20 ng/mL) using Tukey’s HSD test.

## Abbreviations

2-DG2	2-Deoxy-D-glucose
ADP	Adenosine Diphosphate
ATP	Adenosine Triphosphate
BasalGlycoPER	Basal Glycolytic Proton Efflux Rate
CCT	CTP:Phosphocholine Cytidylyltransferase
CDP-choline	Cytidine Diphosphate-Choline
CDP-DAG	Cytidine Diphosphate-Diacylglycerol
CDS2	CDP-Diacylglycerol Synthase 2
CE	Cholesteryl Ester
Cer	Ceramide
CK	Choline Kinase
CL	Cardiolipin
CMP	Cytidine Monophosphate
CPT	CDP-Choline:1,2-Diacylglycerol Cholinephosphotransferase
CTCF	Corrected Total Cell Fluorescence
CTP	Cytidine Triphosphate
DAG or DG	Diacylglycerol
DGAT1	Diacylglycerol O-Acyltransferase 1
DMEM	Dulbecco’s Modified Eagle Medium
DMSO	Dimethyl Sulfoxide
DTT	Dithiothreitol
ECL	Enhanced Chemiluminescence
EMT	Epithelial-to-Mesenchymal Transition
FBS	Fetal Bovine Serum
GAPDH	Glyceraldehyde 3-phosphate dehydrogenase
GlycoPER	Glycolytic Proton Efflux Rate
GPE	Glycerophosphoethanolamine
GPS	Glycerophosphoserine
HCC	Hepatocellular Carcinoma
HexCer	Hexosylceramide
Huh7	Human Hepatoma Cell Line HuH-7
LC-MS	Liquid Chromatography–Mass Spectrometry
LC-MS/MS	Liquid Chromatography–Tandem Mass Spectrometry
LPC	Lysophosphatidylcholine
LPE	Lysophosphatidylethanolamine
LPI	Lysophosphatidylinositol
MAPK	Mitogen-Activated Protein Kinase
MUFA	Monounsaturated Fatty Acids
PA	Phosphatidic Acid
PBS	Phosphate Buffered Saline
PC	Phosphatidylcholine
PCA	Principal Component Analysis
PE	Phosphatidylethanolamine
p-EMT	Partial Epithelial-to-Mesenchymal Transition
PEMT	Phosphatidylethanolamine N-Methyltransferase
PE-O	Ether-Linked Phosphatidylethanolamine
PE-P	Plasmalogen-type Phosphatidylethanolamine
PG	Phosphatidylglycerol
PGS1	Phosphatidylglycerophosphate Synthase 1
PI	Phosphatidylinositol
PI3K/AKT	Phosphoinositide 3-Kinase/Protein Kinase B Pathway
PLD	Phospholipase D
PPi	Inorganic Pyrophosphate
PS	Phosphatidylserine
PSD	Phosphatidylserine Decarboxylase
PTDSS1/2	Phosphatidylserine Synthase 1 and 2
PUFA	Polyunsaturated Fatty Acids
qRT-PCR	Quantitative Reverse Transcription Polymerase Chain Reaction
RNA	Ribonucleic Acid
ROIs	Region of Interest
SCD1	Stearoyl-CoA Desaturase 1
SDS	Sodium Dodecyl Sulfate
Slug	Transcription Factor Slug (SNAI2)
SM	Sphingomyelin
SMADs	Signal Transducers and Transcriptional Modulators Activated by TGF-β Receptors
Snail	Transcription Factor Snail (SNAI1)
TAG or TG	Triacylglycerol
TGF-β	Transforming Growth Factor Beta
TGF-β1	Transforming Growth Factor Beta 1
Twist	Transcription Factor Twist
VIP	Variable Importance in Projection (PLS/OPLS models)
Zeb1	Zinc Finger E-box-Binding Homeobox 1
