# Supplementary material for: Multi-Omic Characterization of Epithelial–Mesenchymal Transition: Lipidomic and Metabolomic Profiles as Key Markers of TGF-β-Induced Transition in Huh7 Hepatocellular Carcinoma
Source: Cells. 2025 Aug 10;14(16):1233. doi: 10.3390/cells14161233 (PMC12384463; doi:10.3390/cells14161233)
Supplement: Supplementary file 1 [file cells-14-01233-s001.zip › cells-3782493-supplementary.pdf]

Supporting material

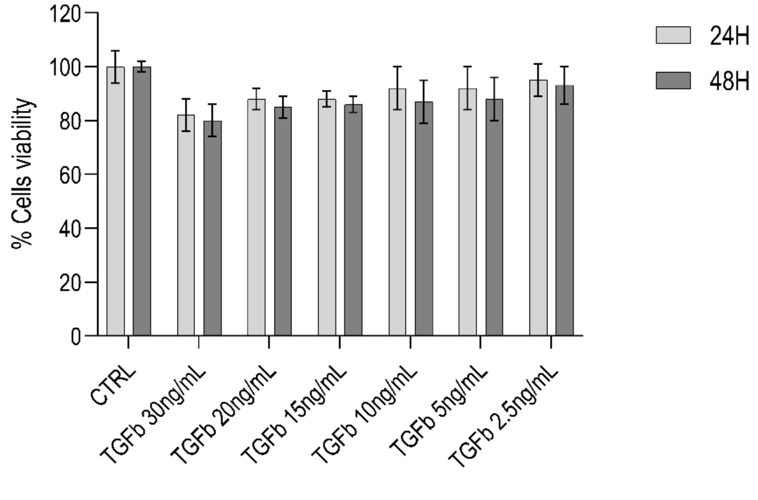

Figure S1. Cell viability assessed by MTT assay at different concentrations of TGF-β. Results are expressed as percentage relative to untreated control cells (CTRL).

Table S1. The table reports the mean ± SD (N = 5) of each lipid species quantified in the lipidomic dataset across three experimental conditions: CTRL, TGF-β1 10 ng/mL, and TGF-β1 20 ng/mL. Lipid abundances were normalized and compared using one-way ANOVA followed by Tukey's HSD post hoc test. For each species, unadjusted and FDR-adjusted p-values are provided. Results of multiple comparisons between experimental conditions (denoted as A, B and C respectively CTRL, TGF-β 10 ng/mL, and TGF-β 20 ng/mL) using Tukey's HSD test.

Species	CTRL (A)	TGF-β1 10ng/mL (B)	TGF-β1 20ng/mL (C)	p_value	FDR	Tukey's HSD
CE 18:1	0.391031 ± 0.040516	0.719935 ± 0.117806	0.894745 ± 0.325804	0.006028567	0.015538419	A-C
CE 18:2	0.177093 ± 0.050379	0.204106 ± 0.103161	0.507645 ± 0.193552	0.002803647	0.00754511	A-C, B-C
CE 20:2	0.408269 ± 0.111135	0.911362 ± 0.155064	1.763015 ± 0.453625	2.52×10^{-5}	0.00012798	A-B, A-C, B-C
CE 22:6	1.285799 ± 0.150296	1.312339 ± 0.61811	1.413153 ± 0.478065			
CE 24:4	0.269541 ± 0.057271	0.077403 ± 0.103343	0.918271 ± 0.734929	0.022944375	0.049397889	B-C
Cer 15:1;2O/15:0	0.000518 ± 5.68E-05	0.000632 ± 2.56E-05	0.000606 ± 8.21E-05	0.025812064	0.05367736	A-B
Cer 17:1;2O/16:0	0.00028 ± 0.000158	0.000417 ± 0.000195	0.000965 ± 0.000106	4.06×10^{-5}	0.000190313	A-C, B-C
Cer 18:1;2O/14:0	0.000793 ± 0.000133	0.000929 ± 0.000118	0.001427 ± 0.000709			
Cer 18:1;2O/16:0	0.007566 ± 0.000696	0.010553 ± 0.000703	0.020059 ± 0.001448	4.92×10^{-10}	9.01×10^{-8}	A-B, A-C, B-C
Cer 18:1;2O/22:0	0.00197 ± 0.00043	0.00293 ± 0.000509	0.004448 ± 0.000509	1.26×10^{-5}	7.01×10^{-5}	A-B, A-C, B-C
Cer 18:1;2O/23:0	0.000985 ± 0.000255	0.001194 ± 0.000159	0.001536 ± 0.000158	0.002708362	0.007509549	A-C, B-C
Cer 18:1;2O/24:0	0.00736 ± 0.001026	0.008407 ± 0.00094	0.009309 ± 0.000831	0.020936516	0.045611694	A-C
Cer 18:2;2O/16:0	0.001613 ± 0.000183	0.002168 ± 0.000266	0.003647 ± 0.00123	0.002527635	0.007146178	A-C, B-C
Cer 18:2;2O/24:0	0.001658 ± 0.000183	0.002258 ± 0.000417	0.001992 ± 0.0002	0.020434593	0.045054585	A-B
Cer 18:2;2O/24:1	0.00031 ± 0.000238	0.000891 ± 0.000469	0.001944 ± 0.000179	1.26×10^{-5}	7.01×10^{-5}	A-B, A-C, B-C
Cer 20:1;2O/24:0	0.000611 ± 0.000342	0.000834 ± 0.000111	0.000735 ± 3.8E-05			

Cer 20:1;2O/24:1	0.000731 ± 0.000103	0.00123 ± 0.000168	0.001597 ± 0.000145	1.99 x 10^{-6}	1.65 x 10^{-5}	A-B, A-C, B-C
CL 16:0_18:1_16:1_18:1	0.010533 ± 0.00217	0.006608 ± 0.000667	0.005556 ± 0.002839	0.006711031	0.016823544	A-B, A-C
CL 16:0_18:1_18:1_18:1	0.003433 ± 0.001024	0.002376 ± 0.000267	0.002748 ± 0.000447			
CL 16:0_18:2_16:1_22:3	0.004156 ± 0.000785	0.003745 ± 0.00053	0.005207 ± 0.000891	0.02570587	0.05367736	B-C
CL 16:1_16:1_16:1_16:1	0.002033 ± 0.000505	0.001682 ± 0.000529	0.002152 ± 0.000191			
CL 16:1_18:1_16:1_18:1	0.03686 ± 0.007765	0.029064 ± 0.002846	0.032254 ± 0.004563			
CL 16:1_18:1_16:1_18:2	0.012603 ± 0.002837	0.011336 ± 0.001167	0.013859 ± 0.001793			
CL 16:1_18:1_18:1_18:1	0.017919 ± 0.00381	0.013957 ± 0.001368	0.015293 ± 0.003121			
CL 16:1_18:1_18:1_18:2	0.017122 ± 0.003466	0.014201 ± 0.001135	0.017312 ± 0.00224			
CL 18:1_18:1_18:1_18:2	0.004524 ± 0.001055	0.003619 ± 0.000692	0.004465 ± 0.000631			
DG 16:0_16:1_0:0	0.049681 ± 0.011938	0.079646 ± 0.042341	0.128743 ± 0.041947	0.012420486	0.02914037	A-C
DG 16:0_18:1_0:0	0.200823 ± 0.015028	0.233872 ± 0.098156	0.235117 ± 0.100392			
DG 16:1_17:1_0:0	0.004609 ± 0.000655	0.006387 ± 0.001218	0.005218 ± 0.002996			
DG 16:1_18:1_0:0	0.134587 ± 0.012176	0.228273 ± 0.048529	0.254356 ± 0.09785	0.027413114	0.05636629	A-C
DG 18:0_18:1_0:0	0.07305 ± 0.008749	0.086323 ± 0.047106	0.132786 ± 0.033176	0.038048092	0.072529174	A-C
DG 18:0_20:2_0:0	0.030009 ± 0.003909	0.030295 ± 0.016249	0.060493 ± 0.016864	0.005773797	0.015313115	A-C, B-C
DG 18:1_18:1_0:0	0.211261 ± 0.038661	0.285713 ± 0.087913	0.352605 ± 0.105788			
DG 18:1_18:2_0:0	0.209217 ± 0.019748	0.242477 ± 0.061306	0.197052 ± 0.025136			
DG 18:1_20:2_0:0	0.089476 ± 0.019348	0.119505 ± 0.039457	0.195943 ± 0.050759	0.002752579	0.007518238	A-C, B-C
HexCer 18:1;2O/16:0	0.000754 ± 3.9E-05	0.001213 ± 0.000116	0.001124 ± 0.000107	1.19 x 10^{-5}	7.01 x 10^{-5}	A-B, A-C
HexCer 18:1;2O/22:0	0.000498 ± 3.22E-05	0.001217 ± 0.000126	0.001083 ± 9E-05	7.00 x 10^{-8}	1.28 x 10^{-6}	A-B, A-C
HexCer 26:1;2O/16:0	0.001045 ± 0.000101	0.001654 ± 0.000244	0.001176 ± 0.000116	0.000215661	0.000857956	A-B, B-C
LPC 18:0	0.028791 ± 0.001311	0.013178 ± 0.00714	0.020193 ± 0.010416	0.018279142	0.040793695	A-B
LPE 18:0	0.009924 ± 0.001192	0.013158 ± 0.0014	0.021419 ± 0.001956	1.90 x 10^{-7}	2.90 x 10^{-6}	A-B, A-C, B-C
LPI 18:0	0.003387 ± 0.000478	0.005377 ± 0.000476	0.008375 ± 0.00078	6.37 x 10^{-8}	1.28 x 10^{-6}	A-B, A-C, B-C
PA 16:0_16:1	0.018443 ± 0.003788	0.011637 ± 0.001276	0.010135 ± 0.00267	0.001082864	0.003669706	A-B, A-C
PA 16:0_18:1	0.021699 ± 0.002139	0.009516 ± 0.001295	0.008764 ± 0.001752	8.30 x 10^{-8}	1.38 x 10^{-6}	A-B, A-C
PA 16:1_18:1	0.015544 ± 0.003474	0.0056 ± 0.001012	0.005082 ± 0.001344	9.82 x 10^{-6}	6.19 x 10^{-5}	A-B, A-C
PC 14:0_14:0	0.109627 ± 0.043472	0.045921 ± 0.023541	0.011498 ± 0.013422	0.00069307	0.002486898	A-B, A-C
PC 14:0_16:0	0.203326 ± 0.111206	0.162591 ± 0.085431	0.046352 ± 0.054793	0.037068662	0.071463867	A-C
PC 14:0_16:1	0.377021 ± 0.180257	0.31374 ± 0.217384	0.389189 ± 0.405968			
PC 14:1_17:1	0.011798 ± 0.005708	0.01156 ± 0.008419	0.009249 ± 0.006366			
PC 15:1_16:0	0.12035 ± 0.083496	0.115827 ± 0.060635	0.088025 ± 0.042121			
PC 16:0_16:0	0.350456 ± 0.118061	0.154801 ± 0.039677	0.274746 ± 0.128781	0.033862351	0.06706083	A-B
PC 16:0_18:0	0.236223 ± 0.117613	0.114138 ± 0.059403	0.107244 ± 0.103385			
PC 16:0_18:1	0.784977 ± 0.317429	0.11849 ± 0.067648	0.136672 ± 0.078115	0.000173683	0.000722362	A-B, A-C
PC 16:0_20:3	0.679669 ± 0.248873	0.464186 ± 0.144413	0.807611 ± 0.604274			
PC 16:0_22:6	0.140042 ± 0.053946	0.152559 ± 0.074553	0.214182 ± 0.095772			

PC 16:1_16:1	0.872882 ± 0.373116	0.499291 ± 0.213189	0.589332 ± 0.270784			
PC 16:1_18:1	0.622091 ± 0.357373	0.452411 ± 0.122725	0.442048 ± 0.235723			
PC 16:1_18:2	0.492955 ± 0.220276	0.398156 ± 0.186395	0.467111 ± 0.313717			
PC 16:1_20:5	0.04308 ± 0.019805	0.034956 ± 0.019615	0.019367 ± 0.016993			
PC 17:0_18:1	0.099989 ± 0.06183	0.065799 ± 0.025318	0.049195 ± 0.016564			
PC 18:0_20:2	0.10873 ± 0.042802	0.104818 ± 0.056707	0.159016 ± 0.072376			
PC 18:0_20:3	0.480398 ± 0.208667	0.487751 ± 0.149129	0.600153 ± 0.194671			
PC 18:1_18:1	0.275246 ± 0.111028	0.259333 ± 0.116364	0.248885 ± 0.097667			
PC 18:1_18:3	0.386075 ± 0.160226	0.325222 ± 0.156939	0.455307 ± 0.338673			
PC 18:1_20:3	0.172731 ± 0.128091	0.132347 ± 0.048711	0.173984 ± 0.086285			
PC 18:1_20:5	0.171794 ± 0.092383	0.152397 ± 0.068937	0.207772 ± 0.094117			
PC 18:2_20:2	0.187225 ± 0.057983	0.152053 ± 0.048294	0.221508 ± 0.11715			
PC 20:3_22:3	0.005475 ± 0.003798	0.006274 ± 0.002747	0.006328 ± 0.004032			
PE 16:0_16:1	0.026824 ± 0.001846	0.029172 ± 0.003698	0.037919 ± 0.004863	0.001131078	0.003763404	A-C, B-C
PE 16:0_18:0	0.002608 ± 0.000282	0.002385 ± 0.000415	0.002345 ± 0.00042			
PE 16:0_18:1	0.076584 ± 0.005207	0.079949 ± 0.009303	0.107327 ± 0.012566	0.000446243	0.001701301	A-C, B-C
PE 16:0_18:3	0.009642 ± 0.00089	0.014971 ± 0.001938	0.015567 ± 0.002412	0.000485401	0.001812826	A-B, A-C
PE 16:0_20:3	0.081381 ± 0.005791	0.114306 ± 0.014584	0.118971 ± 0.017631	0.001737737	0.00538993	A-B, A-C
PE 16:0_20:4	0.080228 ± 0.004991	0.103593 ± 0.013089	0.135823 ± 0.014776	2.92 x 10^{-5}	0.000143442	A-B, A-C, B-C
PE 16:0_20:5	0.015058 ± 0.00123	0.015215 ± 0.00194	0.021735 ± 0.002139	9.46 x 10^{-5}	0.000422044	A-C, B-C
PE 16:0_22:6	0.019955 ± 0.002922	0.042515 ± 0.005507	0.074921 ± 0.007957	1.72 x 10^{-8}	6.30 x 10^{-7}	A-B, A-C, B-C
PE 16:1_18:1	0.071621 ± 0.005357	0.086289 ± 0.011393	0.10276 ± 0.013726	0.002317209	0.006730941	A-C
PE 16:1_18:2	0.007145 ± 0.001031	0.008691 ± 0.001014	0.00724 ± 0.001561			
PE 16:1_20:3	0.026845 ± 0.026031	0.041918 ± 0.034417	0.078445 ± 0.060598			
PE 16:1_20:4	0.010664 ± 0.001411	0.014034 ± 0.001737	0.016244 ± 0.002019	0.000979428	0.0033818	A-B, A-C
PE 18:0_18:1	0.072806 ± 0.007282	0.074721 ± 0.010527	0.102483 ± 0.013167	0.001254261	0.004026837	A-C, B-C
PE 18:0_18:2	0.08044 ± 0.009143	0.085635 ± 0.010578	0.116809 ± 0.016573	0.001224257	0.004000698	A-C, B-C
PE 18:0_20:2	0.015071 ± 0.001626	0.015557 ± 0.002313	0.02243 ± 0.003237	0.000785353	0.002763838	A-C, B-C
PE 18:0_20:3	0.272908 ± 0.018455	0.300271 ± 0.148737	0.429642 ± 0.055974	0.043765214	0.081724838	A-C
PE 18:0_20:4	0.367216 ± 0.078672	0.438893 ± 0.152294	0.61807 ± 0.117051	0.017173479	0.039284334	A-C
PE 18:0_20:5	0.07265 ± 0.031093	0.054816 ± 0.006134	0.079151 ± 0.009032			
PE 18:0_22:3	0.00648 ± 0.000761	0.009155 ± 0.004338	0.024159 ± 0.003282	2.61 x 10^{-6}	2.07 x 10^{-5}	A-C, B-C
PE 18:0_22:6	0.028359 ± 0.002133	0.04309 ± 0.005632	0.070023 ± 0.008614	5.34 x 10^{-7}	5.43 x 10^{-6}	A-B, A-C, B-C
PE 18:1_18:1	0.105913 ± 0.008512	0.093341 ± 0.009298	0.131048 ± 0.0177	0.001512787	0.004773105	A-C, B-C
PE 18:1_18:2	0.082487 ± 0.019663	0.051903 ± 0.023184	0.07647 ± 0.009327			
PE 18:1_20:4	0.115398 ± 0.063753	0.164866 ± 0.021022	0.214927 ± 0.024858	0.008624483	0.021328114	A-C
PE 18:1_22:3	0.006578 ± 0.000885	0.007902 ± 0.004399	0.012553 ± 0.004574			
PE O-16:1_20:3	0.002261 ± 0.000313	0.003286 ± 0.000707	0.004106 ± 0.000779	0.002203346	0.006503426	A-C

PE O-18:1_20:4	0.002483	±	0.000211	0.003191	±	0.000446	0.004581	±	0.000441	5.60 x 10^{-6}	3.80 x 10^{-5}	A-B, A-C, B-C
PE P-16:0_20:4	0.001951	±	0.000149	0.002216	±	0.000291	0.004486	±	0.000545	2.07 x 10^{-7}	2.92 x 10^{-6}	A-C, B-C
PE P-16:0_22:6	0.00292	±	0.00018	0.004489	±	0.00055	0.009375	±	0.000971	6.47 x 10^{-9}	3.94 x 10^{-7}	A-B, A-C, B-C
PE P-18:0_22:6	0.001917	±	0.000152	0.002904	±	0.000491	0.005133	±	0.000581	2.91 x 10^{-7}	3.33 x 10^{-6}	A-B, A-C, B-C
PE P-18:1_20:3	0.001947	±	0.000182	0.002678	±	0.000654	0.004669	±	0.000521	4.52 x 10^{-6}	3.31 x 10^{-5}	A-C, B-C
PE P-20:0_22:6	0.000838	±	8.39E-05	0.00108	±	0.000268	0.002253	±	0.000213	2.54 x 10^{-7}	3.10 x 10^{-6}	A-C, B-C
PG 16:0_18:1	0.037213	±	0.001874	0.029995	±	0.003758	0.017592	±	0.009271	0.000674162	0.002467431	A-C, B-C
PG 16:0_22:6	0.003352	±	0.000294	0.003652	±	0.000296	0.006613	±	0.000899	1.57 x 10^{-6}	1.37 x 10^{-5}	A-C, B-C
PG 16:1_18:1	0.00642	±	0.000936	0.004262	±	0.000563	0.005798	±	0.001699	0.034080094	0.06706083	A-B
PG 16:1_22:6	0.004314	±	0.000667	0.004731	±	0.000671	0.00702	±	0.000999	0.000330982	0.001288718	A-C, B-C
PG 18:0_18:1	0.01163	±	0.002316	0.00619	±	0.000772	0.006152	±	0.001382	0.000193739	0.00078787	A-B, A-C
PG 18:1_18:1	0.017366	±	0.001764	0.021619	±	0.002255	0.021856	±	0.003691			
PG 18:1_18:2	0.008731	±	0.000738	0.013161	±	0.00148	0.015656	±	0.002583	0.000165073	0.000702519	A-B, A-C
PG 18:1_20:3	0.005688	±	0.000915	0.011947	±	0.00146	0.017633	±	0.00316	4.15 x 10^{-6}	3.16 x 10^{-5}	A-B, A-C, B-C
PG 18:1_22:6	0.023958	±	0.002076	0.025363	±	0.003002	0.03928	±	0.004164	9.69 x 10^{-6}	6.19 x 10^{-5}	A-C, B-C
PG 18:2_22:6	0.005214	±	0.000433	0.006598	±	0.000799	0.011772	±	0.00132	2.46 x 10^{-7}	3.10 x 10^{-6}	A-C, B-C
PG 20:3_22:6	0.003776	±	0.000659	0.006783	±	0.000772	0.014309	±	0.001607	1.05 x 10^{-8}	4.79 x 10^{-7}	A-B, A-C, B-C
PG 22:6_22:6	0.011991	±	0.001204	0.00989	±	0.001232	0.023158	±	0.002377	6.53 x 10^{-8}	1.28 x 10^{-6}	A-C, B-C
PI 16:0_18:3	0.015223	±	0.000705	0.016629	±	0.002006	0.015195	±	0.002213			
PI 16:0_20:3	0.152363	±	0.007822	0.158713	±	0.015312	0.156126	±	0.01751			
PI 16:0_20:4	0.053574	±	0.003623	0.043291	±	0.005385	0.045966	±	0.004808	0.011980391	0.028472877	A-B
PI 16:0_22:6	0.013218	±	0.001142	0.008494	±	0.000987	0.009507	±	0.001013	2.98 x 10^{-5}	0.000143442	A-B, A-C
PI 17:0_20:3	0.044953	±	0.002976	0.047503	±	0.005284	0.054466	±	0.004601	0.013627398	0.031567263	A-C
PI 18:0_18:1	0.003748	±	0.001964	0.010314	±	0.000811	0.016093	±	0.002036	3.33 x 10^{-7}	3.58 x 10^{-6}	A-B, A-C, B-C
PI 18:0_18:2	0.051988	±	0.00444	0.061906	±	0.007949	0.067883	±	0.007583	0.010189676	0.024535666	A-C
PI 18:0_20:3	0.595496	±	0.062125	0.648587	±	0.05091	0.744757	±	0.076375	0.009711878	0.023696983	A-C
PI 18:0_20:4	0.461794	±	0.030528	0.378823	±	0.035838	0.450932	±	0.04002	0.006223021	0.015816846	A-B, B-C
PI 18:0_22:3	0.015948	±	0.001279	0.016653	±	0.002077	0.016952	±	0.002149			
PI 18:0_22:6	0.011569	±	0.001014	0.011207	±	0.000935	0.015408	±	0.001184	5.68 x 10^{-5}	0.000259998	A-C, B-C
PI 18:1_18:1	0.036366	±	0.002112	0.031388	±	0.002232	0.035976	±	0.003231	0.017435968	0.039392372	A-B, B-C
PI 18:1_18:2	0.155745	±	0.002916	0.12882	±	0.068892	0.156129	±	0.01751			
PI 18:1_20:3	0.462842	±	0.03097	0.37929	±	0.035554	0.450406	±	0.039421	0.006013516	0.015538419	A-B, B-C

PI 18:1_20:4	0.090595 ± 0.00684	0.04582 ± 0.005357	0.045375 ± 0.004343	2.20 x 10^{-8}	6.72 x 10^{-7}	A-B, A-C
PI 18:1_22:3	0.01922 ± 0.001638	0.012881 ± 0.000937	0.013188 ± 0.001583	1.65 x 10^{-5}	8.64 x 10^{-5}	A-B, A-C
PI 18:1_22:6	0.005914 ± 0.000396	0.003538 ± 0.000612	0.004041 ± 0.000397	1.11 x 10^{-5}	6.79 x 10^{-5}	A-B, A-C
PI 22:1_17:2	0.013764 ± 0.002164	0.013086 ± 0.00061	0.015882 ± 0.001601	0.041699846	0.078670844	B-C
PS 16:0_16:1	0.173886 ± 0.011625	0.121424 ± 0.013103	0.111521 ± 0.014225	1.31 x 10^{-5}	7.04 x 10^{-5}	A-B, A-C
PS 16:0_20:3	0.063767 ± 0.006113	0.063627 ± 0.005141	0.057931 ± 0.006453			
PS 16:0_20:4	0.025713 ± 0.002446	0.023491 ± 0.002864	0.027521 ± 0.004399			
PS 16:0_22:6	0.013775 ± 0.000805	0.015611 ± 0.001733	0.025901 ± 0.003533	5.11 x 10^{-6}	3.60 x 10^{-5}	A-C, B-C
PS 16:1_18:1	0.111438 ± 0.007818	0.061872 ± 0.007001	0.050351 ± 0.000709	4.29 x 10^{-9}	3.93 x 10^{-7}	A-B, A-C, B-C
PS 18:0_16:1	0.388866 ± 0.022746	0.250139 ± 0.023629	0.253594 ± 0.028438	1.54 x 10^{-6}	1.37 x 10^{-5}	A-B, A-C
PS 18:0_18:1	0.212414 ± 0.01726	0.10357 ± 0.012199	0.109827 ± 0.022467	6.26 x 10^{-7}	6.02 x 10^{-6}	A-B, A-C
PS 18:0_18:2	0.223118 ± 0.01441	0.121871 ± 0.012872	0.122682 ± 0.013981	6.19 x 10^{-8}	1.28 x 10^{-6}	A-B, A-C
PS 18:0_20:3	0.321807 ± 0.040137	0.303019 ± 0.030439	0.335585 ± 0.046454			
PS 18:0_22:5	0.061222 ± 0.003706	0.051859 ± 0.005142	0.061798 ± 0.007104	0.024256197	0.05161493	A-B, B-C
PS 18:0_22:6	0.14472 ± 0.009105	0.158738 ± 0.013988	0.210308 ± 0.02382	0.000116428	0.000507292	A-C, B-C
PS 18:1_20:3	0.138817 ± 0.014344	0.147505 ± 0.013746	0.183156 ± 0.019207	0.00204208	0.006228344	A-C, B-C
PS 18:1_20:4	0.044679 ± 0.003496	0.033287 ± 0.004326	0.040787 ± 0.004261	0.00253826	0.007146178	A-B, B-C
SM 16:1;O2_16:0	0.147555 ± 0.026538	0.104026 ± 0.062401	0.024597 ± 0.028462	0.002089317	0.006267951	A-C, B-C
SM 16:1;O2_18:1	0.248635 ± 0.143094	0.19604 ± 0.115596	0.180363 ± 0.16064			
SM 16:1;O2_22:0	0.011164 ± 0.001814	0.013658 ± 0.007035	0.031968 ± 0.019269	0.033566041	0.06706083	A-C
SM 16:1;O2_24:1	0.039407 ± 0.018497	0.063843 ± 0.027363	0.111516 ± 0.055218	0.028237044	0.057415322	A-C
SM 16:1;O2_26:2	0.002815 ± 0.002036	0.003752 ± 0.001885	0.003564 ± 0.002331			
SM 18:0;O2_16:0	0.109822 ± 0.093971	0.073038 ± 0.030178	0.071421 ± 0.052837			
SM 18:0;O2_22:0	0.047159 ± 0.016226	0.040191 ± 0.015577	0.028387 ± 0.010992			
SM 18:0;O2_24:4	0.00563 ± 0.003243	0.007083 ± 0.003364	0.012105 ± 0.007946			
SM 18:1;O2_14:0	0.135452 ± 0.100304	0.117268 ± 0.090658	0.02617 ± 0.028269			
SM 18:1;O2_18:0	0.013143 ± 0.005481	0.016627 ± 0.00468	0.029536 ± 0.032022			
SM 18:1;O2_20:0	0.016816 ± 0.009505	0.036821 ± 0.020838	0.068026 ± 0.048649			
SM 18:1;O2_24:0	0.276444 ± 0.131385	0.200246 ± 0.099745	0.369454 ± 0.17342			
SM 18:1;O2_24:1	0.33946 ± 0.189619	0.282945 ± 0.117525	0.457443 ± 0.30513			
TG 12:0_16:0_16:1	0.109655 ± 0.02479	0.257644 ± 0.155453	0.383485 ± 0.388862			
TG 12:0_16:1_16:2	0.009786 ± 0.002016	0.042996 ± 0.023548	0.044414 ± 0.042927			
TG 14:0_14:0_16:0	0.055298 ± 0.014896	0.07225 ± 0.040461	0.126215 ± 0.145823			
TG 14:0_14:1_16:1	0.058442 ± 0.017297	0.188822 ± 0.083708	0.220529 ± 0.19275			
TG 14:0_16:0_16:1	0.256085 ± 0.063162	0.567338 ± 0.287958	0.797382 ± 0.910859			
TG 14:0_16:0_18:1	0.275643 ± 0.150855	0.246025 ± 0.206652	0.264578 ± 0.260043			
TG 14:0_16:1_18:1	0.732891 ± 0.220896	1.056798 ± 0.564083	1.721949 ± 1.863513			
TG 14:0_16:1_18:2	0.432889 ± 0.162985	1.321744 ± 0.698329	1.896672 ± 2.047547			
TG 14:1_16:0_16:1	0.331261 ± 0.07856	0.88172 ± 0.436903	1.221411 ± 1.160504			
TG 14:1_16:1_16:1	0.101852 ± 0.026024	0.384077 ± 0.202643	0.476466 ± 0.416585			

TG 14:1_16:1_18:2	0.083524 ± 0.023073	0.372864 ± 0.169302	0.54667 ± 0.553635			
TG 15:0_16:0_17:0	0.111142 ± 0.038297	0.11211 ± 0.042479	0.129817 ± 0.139004			
TG 15:0_16:0_18:1	0.066742 ± 0.029484	0.146192 ± 0.06655	0.148128 ± 0.19442			
TG 16:0_16:0_18:0	0.185157 ± 0.042855	0.179696 ± 0.172478	0.277699 ± 0.253126			
TG 16:0_16:1_17:1	0.141609 ± 0.039504	0.263757 ± 0.131039	0.209136 ± 0.193423			
TG 16:0_17:1_18:1	0.151632 ± 0.057226	0.157224 ± 0.164405	0.26973 ± 0.34519			
TG 16:0_18:1_18:1	0.508229 ± 0.079855	0.528266 ± 0.305909	0.427068 ± 0.385171			
TG 16:0_18:1_18:2	1.389343 ± 0.35214	1.943963 ± 0.722969	2.915647 ± 3.289372			
TG 16:0_18:1_22:3	0.339737 ± 0.085071	0.653346 ± 0.383075	1.047666 ± 1.029789			
TG 16:0_18:1_22:6	0.237739 ± 0.050122	0.241839 ± 0.103318	0.504161 ± 0.502663			
TG 16:0_18:1_24:4	0.11425 ± 0.043658	0.171421 ± 0.117671	0.460849 ± 0.472741			
TG 16:1_17:1_18:1	0.095681 ± 0.021381	0.213528 ± 0.100416	0.165515 ± 0.143384			
TG 16:1_18:1_18:2	0.362143 ± 0.080644	1.191852 ± 0.55525	1.116359 ± 0.817521			
TG 16:1_18:1_22:6	0.087788 ± 0.032119	0.145626 ± 0.071523	0.385584 ± 0.405639			
TG 18:0_18:1_18:1	0.522947 ± 0.175997	0.684148 ± 0.681042	1.303393 ± 1.077776			
TG 18:1_18:1_18:2	0.543688 ± 0.175442	0.896882 ± 0.386272	1.401045 ± 1.603456			
TG 18:1_18:1_22:6	0.069329 ± 0.011555	0.09318 ± 0.0447	0.130831 ± 0.115333			
TG 18:1_18:1_24:4	0.028933 ± 0.005958	0.04005 ± 0.041182	0.146611 ± 0.134768			
TG 18:1_18:2_18:2	0.235106 ± 0.054712	0.49452 ± 0.214156	0.466265 ± 0.377262			

Table S2. The table reports the mean ± SE (N = 6) of the normalized peak areas (median centering, log transformation, and autoscaling) of metabolic LC/MS dataset. For each variable the compared condition pairs, unadjusted p-values, and FDR-adjusted p-values are reported. Statistically significant differences (FDR < 0.05) indicate lipid alterations associated with TGF-β treatment. Results of multiple comparisons between experimental conditions (denoted as A, B and C respectively CTRL, TGF-β 10 ng/mL, and TGF-β 20 ng/mL) using Tukey's HSD test.

Variable	CTRL (A)	TGF-β 10ng/mL (B)	TGF-β 20ng/mL (C)	p.value	FDR	Tukey's HSD
(1-Methyl-1H-imidazol-4-yl) acetic acid	1.349315 ± 0.141174	-0.56495 ± 0.167799	-0.78436 ± 0.214148	2.04 x 10^{-12}	3.14 x 10^{-11}	B-A; C-A
(R)-Butyrylcarnitine	1.269497 ± 0.309855	-0.66498 ± 0.603365	-0.60452 ± 0.191904	5.44 x 10^{-7}	1.59 x 10^{-6}	B-A; C-A
α.-D-Galactosamine 1-phosphate	0.895182 ± 0.446731	-0.36854 ± 1.280096	-0.52664 ± 0.323044	0.015022	0.018854	B-A; C-A
α.-D-Galactosamine 1-phosphate2	1.245428 ± 0.228028	-0.39659 ± 0.633008	-0.84883 ± 0.18063	4.56 x 10^{-7}	1.40 x 10^{-6}	B-A; C-A
αD-Galactose 1-phosphate	1.313325 ± 0.226962	-0.53954 ± 0.246566	-0.77379 ± 0.387415	4.53 x 10^{-9}	2.14 x 10^{-8}	B-A; C-A
β.-Cyano-L-alanine	-1.26031 ± 0.465342	0.59338 ± 0.524138	0.666927 ± 0.215449	9.84 x 10^{-7}	2.63 x 10^{-6}	B-A; C-A
1-Methyladenosine	-1.24809 ± 0.55964	0.516578 ± 0.408806	0.731511 ± 0.296735	1.49 x 10^{-6}	3.90 x 10^{-6}	B-A; C-A
1-Methyl-L-histidine	-0.81321 ± 0.315755	-0.06072 ± 1.075782	0.873926 ± 0.654601	0.005181	0.0071599	C-A
1-Methylnicotinamide cation	-1.35931 ± 0.138811	0.57838 ± 0.159064	0.780926 ± 0.070236	1.67 x 10^{-14}	4.12 x 10^{-13}	B-A; C-A; C-B
2,3-Dihydroxypropyl dihydrogen phosphate	1.092612 ± 0.109459	-0.30141 ± 0.734507	-0.7912 ± 0.745482	0.000222	0.00039512	B-A; C-A
2-Hydroxypalmitic acid	-1.22749 ± 0.584465	0.453974 ± 0.401037	0.773514 ± 0.352421	3.10 x 10^{-6}	7.78 x 10^{-6}	B-A; C-A
3-Hydroxybutyrylcarnitine	1.019412 ± 0.507336	-0.75309 ± 0.993731	-0.26632 ± 0.377334	0.001203	0.0018964	B-A; C-A
5-(3-Phenylpropyl)-1H-1,2,3,4-tetrazole	-1.26815 ± 0.401753	0.438506 ± 0.400256	0.829646 ± 0.303013	1.37 x 10^{-7}	4.44 x 10^{-7}	B-A; C-A

5'-S-Methyl-5'-thioadenosine	1.189905 ± 0.720567	-0.58032 ± 0.461704	-0.60959 ± 0.344274	3.08×10^{-5}	6.52×10^{-5}	B-A; C-A
Acetyl-DL-carnitine	-1.22554 ± 0.529762	0.340795 ± 0.4297	0.884746 ± 0.231674	7.54×10^{-7}	2.16×10^{-6}	B-A; C-A
Acetyl-L-threonine	1.008771 ± 0.810375	-0.703 ± 0.833639	-0.30577 ± 0.349212	0.001891	0.0028371	B-A; C-A
Adenosine 5'-diphosphoribose	-1.17411 ± 0.836284	0.470724 ± 0.324224	0.703388 ± 0.285759	4.17×10^{-5}	8.55×10^{-5}	B-A; C-A
Adenosine 5'-monophosphate	-1.21803 ± 0.631424	0.314738 ± 0.222666	0.903292 ± 0.27095	7.84×10^{-7}	2.19×10^{-6}	B-A; C-A
Adenylosuccinic acid	0.632701 ± 0.571879	0.433689 ± 0.418588	-1.06639 ± 0.909414	0.000873	0.0014319	C-A; C-B
Ala-Gln	-1.27173 ± 0.360602	0.445151 ± 0.357497	0.826578 ± 0.379132	1.10×10^{-7}	3.66×10^{-7}	B-A; C-A
Ala-Gln2	-1.3319 ± 0.214187	0.491967 ± 0.171329	0.839929 ± 0.242942	2.97×10^{-11}	2.29×10^{-10}	B-A; C-A; C-B
Ala-Gly	0.698241 ± 0.887785	-0.95638 ± 0.855198	0.258139 ± 0.343317	0.004169	0.0058944	B-A; C-B
Ala-Lys	1.066076 ± 0.427961	-0.50298 ± 0.970452	-0.5631 ± 0.476624	0.000992	0.0015845	B-A; C-A
Arg-Ala	1.066867 ± 0.920057	-0.53036 ± 0.588756	-0.53651 ± 0.397579	0.000987	0.0015845	B-A; C-A
Argininosuccinic acid	1.31652 ± 0.298793	-0.58261 ± 0.329538	-0.73391 ± 0.261894	5.08×10^{-9}	2.30×10^{-8}	B-A; C-A
Cholesterol 3-sulfate	-1.34238 ± 0.198677	0.652494 ± 0.184043	0.689888 ± 0.286768	8.99×10^{-11}	6.50×10^{-10}	B-A; C-A
Choline cation	-1.35766 ± 0.082838	0.51471 ± 0.063786	0.842945 ± 0.081336	7.00×10^{-18}	4.31×10^{-16}	B-A; C-A; C-B
Citric acid	-1.14241 ± 0.971703	0.493807 ± 0.29022	0.648601 ± 0.089513	0.000135	0.0002517	B-A; C-A
Creatine	1.284697 ± 0.12556	-0.31327 ± 0.28548	-0.97143 ± 0.268384	1.69×10^{-10}	1.04×10^{-9}	B-A; C-A; C-B
Creatinine	1.322699 ± 0.194353	-0.57469 ± 0.39446	-0.74801 ± 0.198605	1.85×10^{-9}	9.88×10^{-9}	B-A; C-A
Cys-Gly	-1.1581 ± 0.583961	0.577357 ± 0.537911	0.580746 ± 0.596213	9.28×10^{-5}	0.0001783	B-A; C-A
Cytidine 5'-diphosphocholine	1.215073 ± 0.497271	-0.47616 ± 0.526976	-0.73891 ± 0.419611	7.20×10^{-6}	1.67×10^{-5}	B-A; C-A
Cytidine 5'-monophosphate	-1.24726 ± 0.532511	0.333512 ± 0.193278	0.913745 ± 0.277313	1.03×10^{-7}	3.51×10^{-7}	B-A; C-A; C-B
Cytidine	1.341745 ± 0.120555	-0.55032 ± 0.312281	-0.79142 ± 0.112097	1.70×10^{-11}	1.49×10^{-10}	B-A; C-A
D-Fructose 6-phosphate	0.962867 ± 1.208002	-0.35255 ± 0.300849	-0.61031 ± 0.37624	0.005317	0.0072659	B-A; C-A
D-Fructose	-1.29412 ± 0.290612	0.645179 ± 0.431771	0.64894 ± 0.338477	8.10×10^{-8}	3.02×10^{-7}	B-A; C-A
DL-Ornithine	0.856277 ± 0.824742	-0.13596 ± 0.839475	-0.72031 ± 0.700304	0.011535	0.014779	C-A
DL-Phenylalanine	-0.9505 ± 0.934789	0.13642 ± 0.754115	0.814076 ± 0.236124	0.002142	0.0031744	B-A; C-A
Gln-Gln	1.123061 ± 0.588431	-0.40753 ± 0.420231	-0.71553 ± 0.741595	0.000175	0.00031639	B-A; C-A
Glu-Arg	1.09957 ± 0.420872	-0.61747 ± 0.850353	-0.4821 ± 0.558996	0.000439	0.00077016	B-A; C-A
Glu-Gly-Arg	1.35998 ± 0.121494	-0.71286 ± 0.216819	-0.64712 ± 0.080331	1.86×10^{-13}	3.26×10^{-12}	B-A; C-A
Glycerophosphocholine	-1.32827 ± 0.108762	0.444725 ± 0.289072	0.883546 ± 0.115426	6.11×10^{-12}	5.78×10^{-11}	B-A; C-A; C-B
Guanidine	-0.83841 ± 0.456517	0.069657 ± 0.825199	0.768757 ± 0.97576	0.010061	0.013026	C-A

Guanosine 5'-monophosphate	0.983457 ± 1.090326	-0.46803 ± 0.342423	-0.51542 ± 0.593001	0.004573	0.0063923	B-A; C-A
His-Ala	1.148785 ± 0.284526	-0.51858 ± 0.868535	-0.6302 ± 0.426314	0.000117	0.00022181	B-A; C-A
His-Gly	-1.04196 ± 0.497054	0.408347 ± 0.845506	0.633618 ± 0.673325	0.001397	0.0021756	B-A; C-A
Hypoxanthine	0.774432 ± 0.577448	-1.1616 ± 0.495674	0.387163 ± 0.549661	4.00 x 10^{-5}	8.34 x 10^{-5}	B-A; C-B
Inosine 5'-monophosphate	1.058034 ± 0.638495	-1.12392 ± 0.097026	0.065884 ± 0.344306	9.56 x 10^{-7}	2.61 x 10^{-6}	B-A; C-A; C-B
Inosine	1.128625 ± 0.553047	-1.00036 ± 0.321147	-0.12826 ± 0.491775	4.12 x 10^{-6}	1.01 x 10^{-5}	B-A; C-A; C-B
L-β-Homoserine	-1.34838 ± 0.201469	0.571356 ± 0.220086	0.777026 ± 0.113807	3.78 x 10^{-12}	4.65 x 10^{-11}	B-A; C-A
Lactose	-1.1628 ± 0.346698	0.231027 ± 0.597928	0.931777 ± 0.440286	5.21 x 10^{-6}	1.23 x 10^{-5}	B-A; C-A
L-Arginine	-0.97581 ± 0.90024	0.184106 ± 0.662906	0.791706 ± 0.463294	0.001806	0.0027418	B-A; C-A
L-Aspartic acid	-1.32362 ± 0.392273	0.655381 ± 0.171648	0.668237 ± 0.251047	2.82 x 10^{-9}	1.45 x 10^{-8}	B-A; C-A
L-Carnitine	-1.03261 ± 0.309982	0.213752 ± 0.711108	0.81886 ± 0.811927	0.000588	0.00099043	B-A; C-A
L-Citrulline	1.160949 ± 0.280345	-0.71873 ± 0.868682	-0.44222 ± 0.308024	5.90 x 10^{-5}	0.00011895	B-A; C-A
L-Cystathionine	1.325061 ± 0.102719	-0.38843 ± 0.165596	-0.93663 ± 0.145943	6.46 x 10^{-14}	1.32 x 10^{-12}	B-A; C-A; C-B
L-Glutamic acid	-1.34837 ± 0.200821	0.568083 ± 0.218678	0.780289 ± 0.110635	3.36 x 10^{-12}	4.59 x 10^{-11}	B-A; C-A
L-Glutamine	-1.2961 ± 0.280755	0.87575 ± 0.356327	0.420347 ± 0.214495	3.33 x 10^{-9}	1.64 x 10^{-8}	B-A; C-A; C-B
L-Glutathione, reduced	-1.15966 ± 0.612203	0.613152 ± 0.529007	0.546507 ± 0.567485	8.65 x 10^{-5}	0.00016889	B-A; C-A
L-Leucine	-1.03182 ± 0.648016	0.209184 ± 0.85051	0.822635 ± 0.33844	0.000577	0.0009855	B-A; C-A
L-Lysine	-0.7688 ± 0.970508	-0.19899 ± 0.624016	0.967787 ± 0.433601	0.002378	0.0034411	C-A; C-B
L-Methionine	-1.07596 ± 0.578643	0.052608 ± 0.610975	1.023355 ± 0.206446	1.20 x 10^{-5}	2.69 x 10^{-5}	B-A; C-A; C-B
L-Pipecolic acid	-0.83194 ± 0.874841	-0.18497 ± 0.587767	1.016915 ± 0.420274	0.000687	0.0011417	C-A; C-B
L-Propionylcarnitine	-0.85354 ± 0.416424	-0.22927 ± 0.79967	1.082813 ± 0.492763	0.000155	0.00028463	C-A; C-B
L-Saccharopine	1.10523 ± 0.737553	-0.23556 ± 0.38738	-0.86967 ± 0.515688	7.60 x 10^{-5}	0.0001508	B-A; C-A
L-Serine	1.348176 ± 0.229229	-0.57858 ± 0.211861	-0.7696 ± 0.095045	5.22 x 10^{-12}	5.35 x 10^{-11}	B-A; C-A
L-Threonine	-1.32411 ± 0.23994	0.524997 ± 0.368938	0.799108 ± 0.073297	5.71 x 10^{-10}	3.19 x 10^{-9}	B-A; C-A
L-Tyrosine	-1.25965 ± 0.416557	0.380382 ± 0.422677	0.879269 ± 0.205898	9.66 x 10^{-8}	3.39 x 10^{-7}	B-A; C-A
Lys-Gly	1.090271 ± 0.530309	-0.68277 ± 0.92101	-0.4075 ± 0.292237	0.000445	0.00077016	B-A; C-A
Lys-Ile	1.332846 ± 0.298572	-0.80299 ± 0.154003	-0.52985 ± 0.211456	9.89 x 10^{-11}	6.76 x 10^{-10}	B-A; C-A
Methylmalonic acid	-0.83516 ± 0.45195	-0.3682 ± 0.479453	1.203361 ± 0.47801	4.71 x 10^{-6}	1.14 x 10^{-5}	C-A; C-B
N-(Hydroxymethyl) nicotinamide	0.848845 ± 1.213258	-0.80167 ± 0.34908	-0.04717 ± 0.409406	0.007211	0.0096401	B-A

N8-Acetylspermidine	1.3708 ± 0.080667	-0.63253 ± 0.051421	-0.73827 ± 0.042091	2.00×10^{-19}	2.46×10^{-17}	B-A; C-A; C-B
N-Acetyl-D-galactosamine-6-phosphate	1.301663 ± 0.224579	-0.83653 ± 0.266337	-0.46513 ± 0.382204	5.23×10^{-9}	2.30×10^{-8}	B-A; C-A
N-Acetyl-D-glucosamine	0.839769 ± 1.142888	-0.76812 ± 0.700801	-0.07165 ± 0.205573	0.009984	0.013026	B-A
N-Acetyl-L-aspartic acid	1.36085 ± 0.080066	-0.80229 ± 0.105078	-0.55856 ± 0.116032	4.90×10^{-16}	2.01×10^{-14}	B-A; C-A; C-B
N-Acetyl-L-glutamic acid	-0.97757 ± 0.347866	-0.25418 ± 0.423446	1.231747 ± 0.233982	4.38×10^{-8}	1.68×10^{-7}	B-A; C-A; C-B
N-Acetyltryptophan	-0.74935 ± 0.868464	-0.11171 ± 0.931879	0.861056 ± 0.44595	0.009271	0.012262	C-A
NG,NG-Dimethyl-L-arginine2	0.715889 ± 0.904104	-0.76452 ± 0.860103	0.048628 ± 0.723602	0.025154	0.031252	B-A
Nicotinamide riboside cation	0.92502 ± 0.877549	-0.71849 ± 0.661305	-0.20653 ± 0.703652	0.005587	0.007551	B-A; C-A
Palmitoyl-L-carnitine	-1.33719 ± 0.287375	0.646298 ± 0.211362	0.690894 ± 0.230217	2.71×10^{-10}	1.59×10^{-9}	B-A; C-A
Pantothenic acid	0.763609 ± 0.975354	-0.64041 ± 0.94138	-0.1232 ± 0.59364	0.036908	0.045397	B-A
Phosphocholine	-1.36598 ± 0.150311	0.692023 ± 0.107873	0.673958 ± 0.08334	4.20×10^{-15}	1.29×10^{-13}	B-A; C-A
Pro-Asp	1.270077 ± 0.267976	-0.59754 ± 0.601405	-0.67254 ± 0.244035	5.14×10^{-7}	1.54×10^{-6}	B-A; C-A
Pro-Gly	1.204764 ± 0.546279	-0.6413 ± 0.536781	-0.56346 ± 0.444061	1.66×10^{-5}	3.65×10^{-5}	B-A; C-A
Prolylalanine	0.988977 ± 0.809958	-0.72012 ± 0.595866	-0.26886 ± 0.711475	0.002355	0.0034411	B-A; C-A
Pro-Thr	1.245825 ± 0.170761	-0.5845 ± 0.22622	-0.66133 ± 0.722758	2.32×10^{-6}	5.93×10^{-6}	B-A; C-A
S-(5'-Adenosyl)-L-homocysteine	1.125535 ± 0.342904	-0.19506 ± 0.696123	-0.93048 ± 0.43935	1.85×10^{-5}	3.99×10^{-5}	B-A; C-A
S-Adenosyl-L-methionine	1.282391 ± 0.246031	-0.54816 ± 0.510256	-0.73423 ± 0.313347	1.52×10^{-7}	4.79×10^{-7}	B-A; C-A
Sarcosine	1.280347 ± 0.154138	-0.29614 ± 0.277155	-0.98421 ± 0.254242	1.41×10^{-10}	9.13×10^{-10}	B-A; C-A; C-B
Ser-Lys	1.04226 ± 0.365867	-0.53167 ± 1.03103	-0.51059 ± 0.497496	0.001628	0.0025023	B-A; C-A
S-Lactoylglutathione	-1.3396 ± 0.253751	0.504293 ± 0.148851	0.835307 ± 0.132507	4.41×10^{-12}	4.94×10^{-11}	B-A; C-A; C-B
Spermine	-1.20636 ± 0.841139	0.456093 ± 0.131541	0.750269 ± 0.060851	9.60×10^{-6}	2.19×10^{-5}	B-A; C-A
Taurine	-1.29786 ± 0.311372	0.499076 ± 0.416609	0.798781 ± 0.208833	1.75×10^{-8}	7.16×10^{-8}	B-A; C-A
Thiamine cation	-1.34702 ± 0.206495	0.675949 ± 0.272942	0.671072 ± 0.129665	2.92×10^{-11}	$2.29E \times 10^{-10}$	B-A; C-A
Threonic acid	1.316999 ± 0.263951	-0.66764 ± 0.319728	-0.64936 ± 0.325313	6.93×10^{-9}	2.94×10^{-8}	B-A; C-A
Tyr-His	0.456966 ± 0.933307	-1.00076 ± 0.678997	0.54379 ± 0.510545	0.003389	0.0048466	B-A; C-B
Uridine 5'-diphosphoglucuronic acid	-1.30108 ± 0.513338	0.530888 ± 0.137118	0.770196 ± 0.190379	1.94×10^{-8}	7.70×10^{-8}	B-A; C-A
Uridine 5'-monophosphate	-1.28522 ± 0.526688	0.519566 ± 0.1838	0.765659 ± 0.28149	8.92×10^{-8}	3.23×10^{-7}	B-A; C-A
Xylulose 5-phosphate	0.380913 ± 0.787647	-0.9095 ± 0.578239	0.528583 ± 0.971206	0.012621	0.016005	B-A; C-B

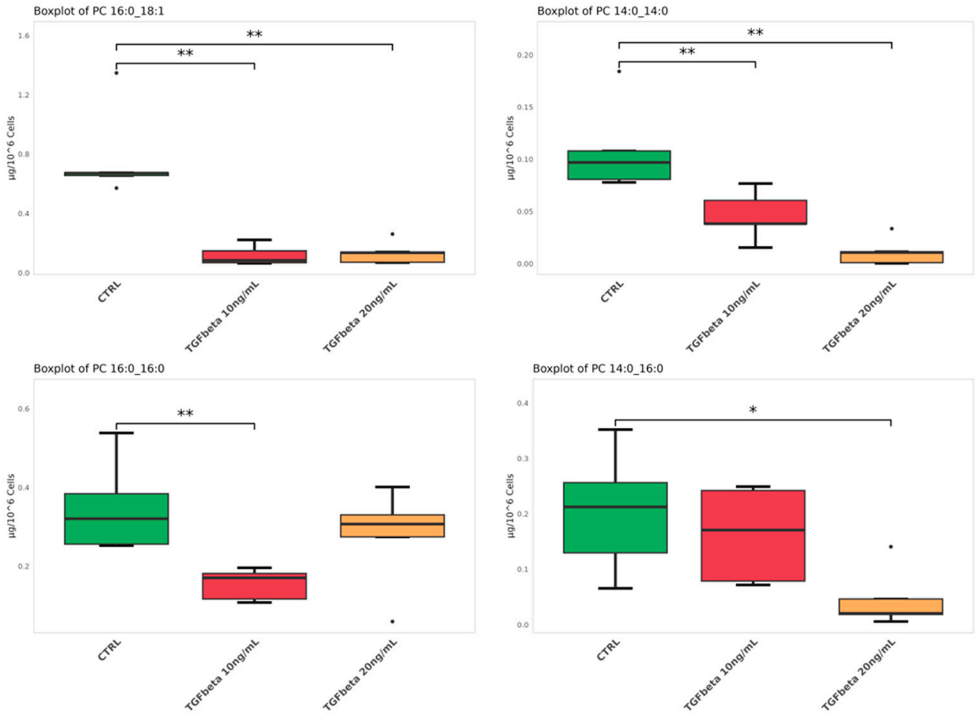

Figure S2. The figure shows four boxplots representing the concentrations (in µg/10^6 cells) of different phosphatidylcholine (PC) species: PC 16:0_18:1 (A); PC 14:0_14:0 (B); PC 16:0_16:0 (C); PC 14:0_16:0 (D). Each boxplot shows the distribution of values with the median (center line), quartiles (boundaries of the box), and outliers (outer dots). ** indicates a statistically significant difference with a p-value < 0.01; * indicates a statistically significant difference with a p-value < 0.05.
